# Systematic discovery of subcellular RNA patterns in the gut epithelium

**DOI:** 10.1186/s13059-025-03786-1

**Published:** 2025-10-29

**Authors:** Minkyoung Lee, Ilhan E. Acar, Davide Eletto, Srivathsan Adivarahan, Farah Mhamedi, Kristina Handler, Jihyun Lee, Elena Guido Vinzoni, Gustavo Aguilar, Andreas E. Moor

**Affiliations:** 1 Department of Biosystems Science and Engineering, ETH Zürich, Schanzenstrasse 44, Basel, 4056 Switzerland; 2 Biozentrum, University of Basel, Spitalstrasse 41, Basel, 4056 Switzerland; 3 Present address: Gene Lay Institute of Immunology and Inflammation, Brigham and Women’s Hospital, Massachusetts General Hospital and Harvard Medical School, Boston, MA USA; 4 Present address: Institute of Experimental Immunology, University of Zürich, Zurich, Switzerland; 5 Present address: Department of Biological Engineering, Massachusetts Institute of Technology, Cambridge, MA USA

**Keywords:** RNA localization, Subcellular transcriptome, APEX-seq, Next-generation sequencing, MERFISH, Spatial transcriptomics, smFISH, Biotinylation, Organoids, Gastrointestinal tissue, RNA granules

## Abstract

**Background:**

Subcellular RNA localization is crucial for the spatio-temporal control of protein synthesis and underlies key processes during development, homeostasis, and disease. In epithelial cells, RNA can localize asymmetrically along the apico-basal axis. Yet, the localization of most transcripts as well as the diversity of patterns that they adopt remains unexplored.

**Results:**

Here, we use APEX-seq for proximity labeling and MERFISH for spatial transcriptomics to map subcellular transcript localization in intestinal organoids and tissue from adult mice. Many transcripts present localization bias, often localizing in granular structures. We uncover intrinsic and environmental factors that influence the formation of these patterns. Additionally, we identify translation-dependent and -independent localization patterns and pinpoint the role of 3′ untranslated regions and RNA-binding proteins.

**Conclusions:**

This subcellular RNA atlas presents a detailed resource for understanding intestinal physiology.

**Supplementary Information:**

The online version contains supplementary material available at 10.1186/s13059-025-03786-1.

## Background

The precise subcellular localization of RNA is a fundamental mechanism underpinning various biological processes, from embryonic development to adult homeostasis [1–3]. In these contexts, spatio-temporal distribution of messenger RNAs regulates the local expression of proteins. However, the extent to which mechanism affects the transcriptome across different cell types remains largely unknown, in part due to the lack of precise mRNA localization charting techniques. Traditionally, most RNA-localization studies have focused on describing one or few mRNA species. Recently, high-throughput studies using proximity labeling [4–7], mechanical isolation [8–11], differential centrifugation [12, 13], or fluorescent based sorting [14] have generated detailed maps of RNA localization in cultured cells. Nevertheless, the extent to which these cultured systems reflect in vivo RNA spatial distribution remains unclear.

We have previously reported that mRNA localizes asymmetrically along the apico-basal axis of enterocytes [2]. Enterocytes are the most abundant cell type in the small intestine, where they mediate the uptake and processing of nutrients during digestion. The apical and basal compartments of enterocytes are highly specialized cellular domains, exposed to radically different environments. The apical milieu faces the intestine’s lumen, where nutrient levels change periodically and cells are in contact with the microbial communities. On the basal side, the intestinal epithelium releases nutrients into the lamina propria, where they enter the circulation. On the other hand, the lamina propria is populated by endothelial, immune, and neural cells. Adding to this complexity, enterocyte properties and environmental conditions vary along their position in the crypt-villus axis [15, 16] and their location along the gut [17].

Our previous observations led us to propose that mRNA localization may serve as a mechanism of environmental adaptation [2]. However, the limited sensitivity of sequencing after laser capture microdissection (LCM-seq) likely resulted in missing many asymmetrically localized RNAs. In this study, we have leveraged the power of proximity-mediated RNA labeling and subcellular spatial transcriptomics to generate a high-resolution map of mRNA localization in both intestinal organoids and intact intestinal tissue. Additionally, we have described the diversity of localization patterns along the villus. Finally, we shed light on the role of translation, the interaction between 3′UTR and RNA-Binding-Proteins (RBPs), and nutrient availability in the formation of these patterns.

## Results

### APEX-seq reveals subcellular transcript localization patterns in intestinal organoids

**Fig. 1:**
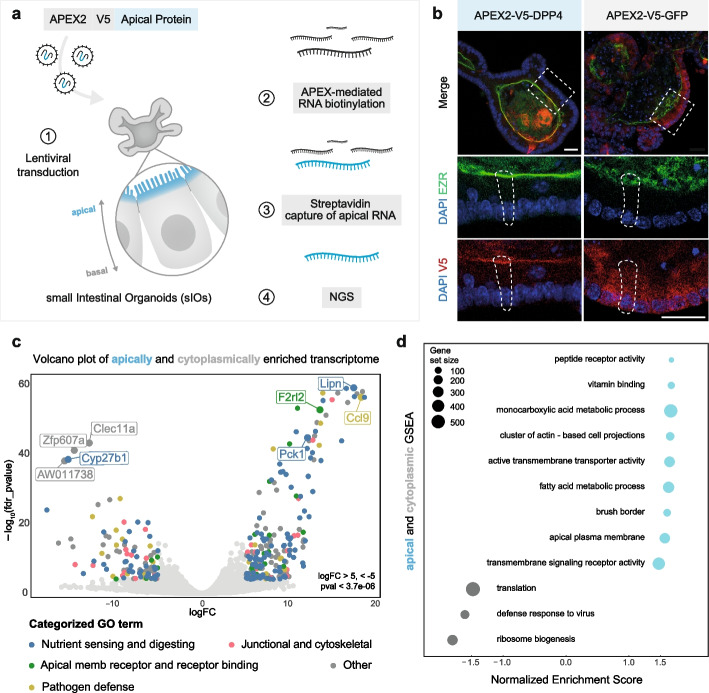
Sequencing-based RNA map with subcellular resolution in small intestinal organoids (sIOs).** a** Schematic representation of the method used for generating the sequencing-based apical RNA map. **b** Immunofluorescence of sIOs with anti-V5 in red, Ezrin (EZR), a marker for the apical membrane, in green and DAPI in blue. White rectangles in top images indicate the location of the insets. Dashed lines in insets indicate outlines of epithelial cells. Scale bar 20µm (5 µm for inset). **c** Volcano plot of the apically enriched transcriptome shown on the right side. Left side shows enriched transcripts in cytoplasm. Transcripts are categorized based on 5 different gene functions: Nutrient sensing and digesting, Apical membrane receptor and receptor binding, Pathogen defense, Junctional and cytoskeletal and Others (Additional Files 1). Genes with more than 5 |log_2_ fold change| (|logFC|) and pFDR < 3.7e − 06 (Bonferroni corrected *p* value threshold) were colored in the plot. **d** Gene Set Enrichment Analysis (GSEA) of apically enriched transcriptome in cyan and cytoplasm in gray (Additional Files 2: Table S3)

In order to capture RNA in its cognate cellular environment, we utilized APEX2, an ascorbate peroxidase that can catalyze the oxidation of biotin-phenol (BP) in the presence of H_2_O_2_. BP-radicals are then covalently integrated into the G-rich regions of RNAs within approximately 20 nm [4, 5, 18]. Subsequently, biotin-labeled RNAs can be captured using streptavidin and identified by next-generation sequencing (NGS) (Fig. 1a and Methods). So far, APEX-mediated biotinylation of RNA has only been achieved in 2D culture, which poorly reflects apico-basal polarity. Thus, we set out to adapt this technology to small intestinal organoids (sIOs) which are grown as intestinal crypt-villus units in laminin-rich matrigel together with chemical growth factors in media (details in the Methods), a 3D model system that recapitulates the main properties of intestinal tissue architecture, including apico-basal polarity [19]. As a proof of concept, we first targeted APEX2 to the mitochondria using the previously reported MITO-V5-APEX2 fusion protein [20]. Immunofluorescence staining for the V5 tag confirmed mitochondrial targeting, with V5 signal overlapping with TOM20, a well-established mitochondrial marker (Additional Files 1: Fig. S1a). qRT-PCR of biotinylated RNA demonstrated a strong enrichment of the mtDNA-encoded mRNA *Mtnd1* over the cytoplasmic control *Actb* [21] (Additional Files 1: Fig. S1b). Along the same lines, transcriptome-wide sequencing revealed an enrichment of mitochondrial encoded mRNAs in samples treated with H_2_O_2_ versus unexposed controls (Additional Files 1: Fig. S1c). These results demonstrate that APEX-seq can be used in organoids to capture distinct subcellular RNA pools.

To map apically localized transcripts, we fused APEX2 to Dipeptidyl peptidase-4 (DPP4). DPP4 is a transmembrane protein previously shown to present a strict apical membrane localization [22, 23]. SIOs transduced with an APEX2-V5-DPP4 fusion presented apical V5 immunoreactivity (Fig. 1b and Additional Files 1: Fig. S2a,b). To serve as controls, we included two additional fusion proteins, APEX2-V5-GFP [5] and APEX2-V5-ACTB [20], which localized in the cytoplasm (Fig. 1b and Additional Files 1: Fig. S1d). Biotinylation resulted in intended apical or cytoplasmic localization respectively, highlighting the specificity of the methodology (Additional Files 1: Fig. S2c). Biotinylated and high-quality RNAs (RNA integrity score > 8.5) were enriched using streptavidin and sent for identification using next-generation sequencing (Additional Files 1: Fig. S2d,e). Gene set enrichment analysis (GSEA) revealed that differentially expressed genes (DEGs) between APEX2-ACTB and APEX2-GFP were enriched in GO terms related to the cytoskeleton, such as cell-adhesion molecule binding, positive regulation of cell adhesion and cell periphery, which underlined the robustness of APEX-seq (Additional Files 1: Fig. S1e). Yet APEX2-ACTB yielded weaker anti-V5 immunostaining, therefore, APEX2-GFP was used in subsequent analyses (Fig. 1b and Additional Files 1: Fig. S1d).

After initial quality control of the samples (Additional Files 1: Fig. S2f,g and Methods), we performed differential expression analysis to identify genes that were significantly enriched in the APEX2-DPP4 samples (apical side) compared to APEX2-GFP (cytoplasm). Using this approach, 1211 and 1128 transcripts were identified as significantly enriched in apical and cytoplasmic zones, respectively (the positive false discovery rate (pFDR) < 0.05, |log2FC|> 1, out of the total 15,330 transcripts we identified)(Additional Files 2: Table S1). These results indicate that many mRNAs are asymmetrically distributed across the apicobasal axis in epithelial cells. To systematically characterize this apical transcriptome atlas, we categorized the differentially enriched 2339 transcripts into four general categories based on curated gene ontology (GO) terms: (1) Nutrient sensing and digesting, (2) Apical membrane receptor and receptor binding, (3) Pathogen defense, and (4) Junctional and cytoskeletal (Fig. 1c and Additional Files 2: Table S2). The categories “Nutrient sensing and digesting” and “Apical membrane receptor and receptor binding” showed higher ratios in apically enriched transcripts (41% vs 24% and 10% vs 1%, respectively). The other two categories did not present big differences (Additional Files 2: Table S2). We then performed GSEA on the results of differential expression analysis and identified “receptor activity,” several “metabolic processes,” “brush border,” and “apical plasma membrane” as some of the most enriched GO terms among apical transcripts. “Translation,” “defense response to virus,” and “ribosome biogenesis” were some of the most enriched among cytoplasmic transcripts (Fig. 1d, Additional Files 2: Table S3), corroborating our previous study [2], in which we found basal localization of ribosomal protein genes in murine small intestine tissue. These results broadly support the notion that transcript localization correlates with where the encoded protein carries out its function. In addition, we made a comparative analysis on the sequencing-based methods APEX-seq and LCM-seq from Moor et al. [2]. Our results confirm the high sensitivity of APEX-seq and highlight the differences in the targeted areas between the two methods (Additional Files 1: Fig. S2h and Additional Files 2: Table S6). The APEX-seq from this study aimed to isolate the transcripts localized in the most apical area, where DPP4 (a cell surface glycoprotein receptor) is localized. On the other hand, the LCM-seq isolated transcriptome targets a bigger apical area of epithelium. This analysis confirms that our new seq-based methodology maps the subcellular RNA with higher resolution.

### Single molecule RNA FISH uncovers granular RNA patterns

**Fig. 2:**
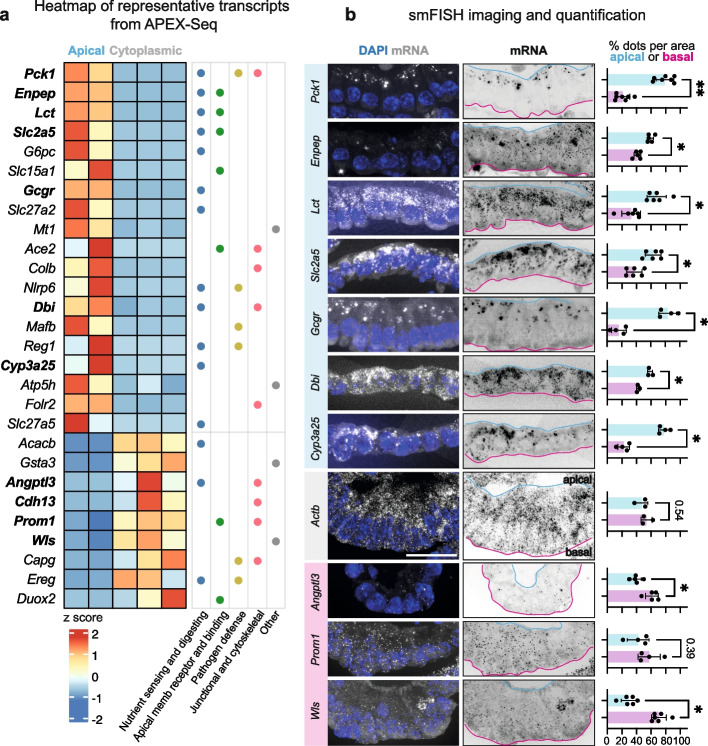
Imaging-based validation of the seq-based RNA map in sIOs.** a** Heatmap indicating the *z* score for localizing transcripts (fdr_pval ≤ 0.05) are listed and annotated with the curated GO term categories (Additional Files 2: Table S7). The bold RNAs indicate the smFISHed RNAs in this paper. Each column represents one replicate. **b** Representative smFISH images of 7 apically and 3 cytoplasmically localizing RNAs. *Actb*-mRNA smFISH image is included as non-localizing control. Apical end of cells is marked with cyan line and the basal end with magenta. DAPI in blue and each transcript dot in white/black. Scale bar 20 µm. Quantification of dots from apical and basal regions, respectively (Additional Files 2: Table S8). Each dot represents a percentage in one measured ROI. Results of ratio paired *t* test are indicated (two-tailed, apical and basal regions were paired in every image). ** *p* val < 0.001, * *p* val < 0.01 and not significant, *p* val is noted

To validate the transcript localization patterns identified using APEX-seq, we performed single-molecule fluorescent in situ hybridization (smFISH) in sIOs [24]. We analyzed a subset of highly enriched RNAs either in APEX2-DPP4 or APEX2-GFP by smFISH imaging and follow-up quantification (Fig. 2a and Additional Files 1: Fig. S3a,b). *Actb*-mRNA, which is evenly distributed throughout the cytoplasm, was included for comparison. The localization of most transcripts coincided with the APEX-seq datasets (Fig. 2b). Of the 13 tested candidates, only *Cdh13*-mRNA did not localize as predicted through APEX-seq, displaying an apical instead of a cytoplasmic localization (Additional Files 1: Fig. S3c,d). Of note, *Cdh13*-mRNA did appear to localize sub-apically, leaving a gap at the very top of the apical region, which may explain the discrepancy, as DPP4 is a membrane protein localizing to the brush border in the apical end of the epithelium (Additional Files 1: Fig. S3d). In comparison, *Apob-*mRNA was highly abundant and localized closely to the apical border (Additional Files 1: Fig. S3d). The smFISH validation experiments indicate that APEX-seq accurately reports on proximity transcriptomics in sIOs.

Many of the transcripts enriched in the apical region presented a granular distribution (Fig. 2b). For instance, *Pck1*, which encodes for a cytosolic gluconeogenic enzyme [25], localized mainly in granular structures. Others, such as the transcript *Lct*, a digestive enzyme involved in lactose digestion [26], were detected in both granular and non-granular patterns. This pattern is not correlated with RNA expression per se. Rather, for the group of 10 RNAs for which we have quantified foci diameters (Additional Files 1: Fig. S4a), we do not observe a positive correlation with expression level which is recorded in our cytoplasmic RNA-seq dataset. Transcripts with granular localization presented with an average spot diameter greater than 0.5 µm, suggesting an aggregation of multiple mRNA copies (Additional Files 1: Fig. S4a). In comparison, the average spot size for *Actb* transcripts was 0.19 µm (Additional Files 1: Fig. S4a). Transcripts of interest with cytoplasmic (*Angptl3*-mRNA, *Prom1*-mRNA) or basal (*Wls*-mRNA) localization did not form notable foci (Fig. 2b).

RNA granules are dynamic structures that play a role in numerous molecular processes, such as active translation, RNA degradation, or silencing [27, 28]. Since many of the apically enriched transcripts are related to nutrient sensing (Figs. 1c and 2a), we speculated that this granular or foci localization pattern may be related to nutrient availability. To test this hypothesis, we starved organoids for 6 h using Earle’s balanced salt solution [29]. In the following 2 h, organoids were either kept in the same media or re-fed using conventional organoid growth media. As an example, we investigated the localization of *Lct*-mRNA, which localizes to the apical region in homeostasis [2] (Fig. 2b). In the starved condition, *Lct*-mRNA was less abundant and localized throughout the cytoplasm without forming foci (Additional Files 1: Fig. S4b-d). Interestingly, the granular apical distribution was re-stored in the re-fed organoids (Additional Files 1: Fig. S4b,d). In addition, we assessed the localization of two RNAs involved in nutrient sensing and translational control: *Mtor* and *Eif4h* [30, 31]*.* We observed that *Mtor* and *Eif4h* showed granular patterns upon the re-feeding that resembled *Lct*. *Mtor*-mRNA were localizing in the apical side of epithelium regardless of nutrient status, but under the starvation, the number of foci was reduced in abundance and size compared to the re-fed condition (Additional Files 1: Fig. S4b,d). Also, *Eif4h*-mRNA showed higher number and larger granular structures in the regained nutrient condition than the nutrient-depleted condition (Additional Files 1: Fig. S4b,d). Based on our observation and quantification, nutrient availability can affect the formation of larger foci and transcript abundance of the genes associated with nutrient intake, sensing, or protein translation. This experiment indicates that the apical mRNA foci of enterocytes are dynamic structures and that can change upon variations in the environmental conditions.

### Translational-dependent and -independent RNA localization in sIOs

**Fig. 3:**
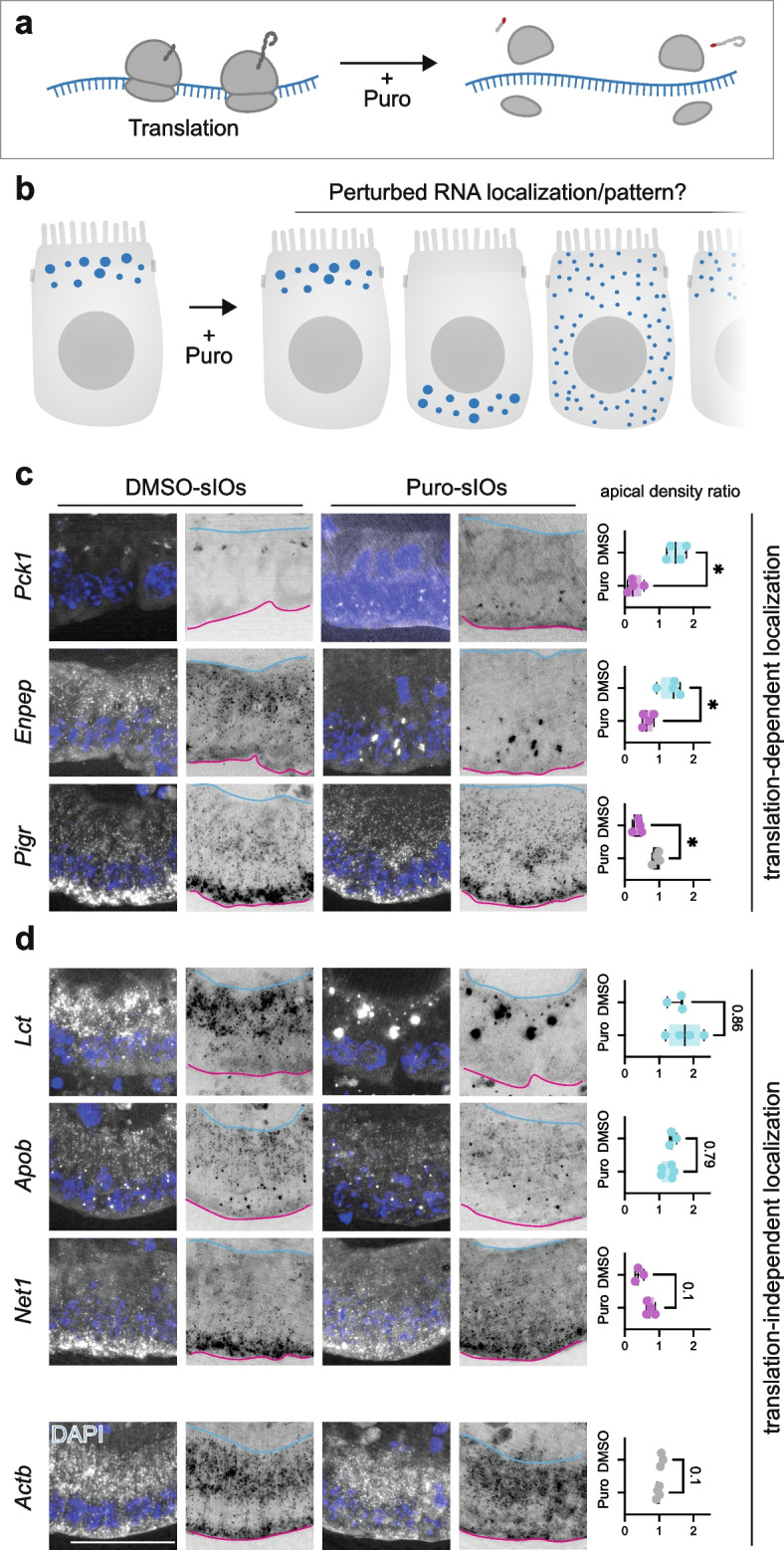
Translational-complex dissociation reveals translational-dependent and -independent RNA localization patterns.** a** Scheme of puromycin treatment causing dissociation of ribosome, mRNA, and nascent peptide. **b** Scheme of possible scenarios upon the external perturbation. RNA may remain unperturbed or change its localization partially or totally. **c,d** Representative smFISH images of indicated transcripts and conditions (left) and corresponding quantifications (right). mRNAs with translation-independent localization: *Lct*-mRNA, *Apob*-mRNA and *Net1*-mRNA. Translation-dependent localization: *Pck1*-mRNA and *Enpep*-mRNA show a localization shift from apical to basal upon puromycin treatment. *Pigr*-mRNA shows less basal RNA localization pattern upon the drug treatment. Apical end of cells is marked with cyan line and the basal side with magenta. DAPI in blue and each transcript dot in white/black. Scale bar 20 µm. Color coding in the graph indicates apical (cyan), basal (magenta), or cytoplasmic (gray) localization ratios. Quantification of dots from apical and basal regions, respectively (Additional Files 2: Table S8). Each dot represents one measured ROI, > 3 images were analyzed for each sample. Significant differences are tested for by unpaired Mann Whitney *t* test. * *p* value < 0.01 and ns = not significant, *p* val is noted

This and previous studies have revealed some of the environmental determinants and molecular players that can influence the localization of RNA in gut cells [2, 3, 7, 32]. To facilitate these localization patterns, it is likely that asymmetrically localizing mRNAs present unique qualities. These differences could be associated with the RNA sequence itself [33] or to the nascent peptide, which is associated with the mRNA/ribosome complex during translation [34]. Given the wide variety of localization patterns uncovered in this study, we hypothesized that both mechanisms may co-exist, driving the localization of different RNAs. To distinguish between them, we first challenged the role of the nascent peptide by dissociating it from the mRNA. We expect this dissociation to result in localization changes of those mRNAs that are mediated by their nascent peptide. To this end, we briefly treated sIOs with puromycin, a well-known inhibitor of translation and inducer of translational complex dissociation [35], and visualized the localization of several transcripts using smFISH (Fig. 3a,b). In accordance with our hypothesis, we observed transcript-specific responses to the inhibitor. While some transcripts, such as *Pigr*-mRNA, presented a mild loss of polarity in their pattern, others, like *Enpep*-mRNA and *Pck1*-mRNA, inverted their polarity, from their original apical localization to the basal region (Fig. 3c). In addition, several of the asymmetrically localized transcripts, for example *Lct*, *Apob*, and *Net1*-mRNAs did not change their localization pattern (Fig. 3d), suggesting that the localization of these mRNAs is mediated by a translation-independent mechanism. Interestingly, puromycin treatment also resulted in formation of large foci, also dose dependently, for *Enpep* and *Lct* (Fig. 3c,d and Additional File 1: Fig. S5a,b). This is not related to oxidative stress based on unchanged in the RNA foci size between DMSO group and sodium arsenite-treated group [36] (Additional Files 1: Fig. S5a,b). When we perturbed translation also with harringtonine, which inhibits initiation of polypeptide synthesis by immobilizing ribosome immediately after translation initiation [37], and cycloheximide, which blocks protein synthesis, specifically interfering with the translation elongation [38], we observed similar granular pattern on *Enpep* and *Lct*-mRNA. *Actb* and *Lct* RNA maintained their localization (Additional File 1: Fig. S5c). Consequently, they are categorized as exhibiting translation-independent localization, highlighting the role of their mRNA sequence in this process. On the other hand, *Enpep* RNA shifted from apical to basal upon puromycin and harringtonine treatment showing its translation-dependent localization but with the cycloheximide treatment, *Enpep* RNA remained its localization in apical side like the control condition forming the granular pattern (Additional Files 1: Fig. S5c). This reflects that translational machinery and nascent peptides are important factors for *Enpep* RNA to localize apically. Together, these results highlight the existence of multiple mechanisms, both translation-dependent and independent, to achieve asymmetric mRNA localization in intestinal epithelial cells.

### RBPs recognizing the 3′UTR mediate apical mRNA localization in sIOs

**Fig. 4:**
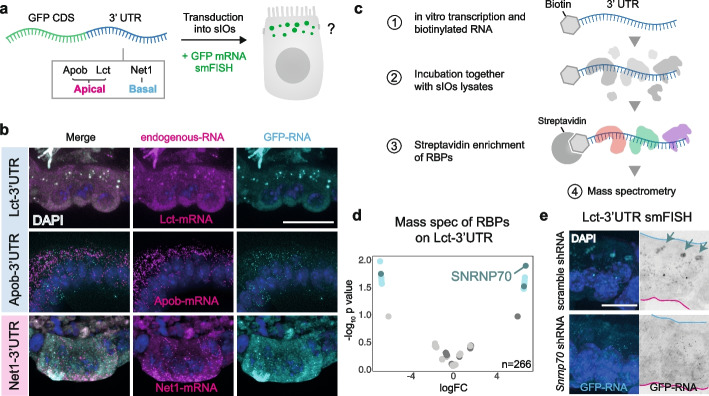
Identifying potential RNA localization signals in 3′UTR and isolating its driving factors.** a** Scheme of introduced RNA candidates under the common GFP coding sequence (CDS). Chimeras containing the 3′UTR of *Lct*,* Apob*, or *Net1* are transfected into sIOs. smFISH probes are used to target the GFP region. **b** Representative smFISH images of indicated constructs. DAPI in blue, endogenous mRNA in magenta and GFP transcripts in cyan. Scale bar 20 µm. **c** Schematic of the RBP mass spectrometry (RBP-MS) experiment. **d** Volcano plot of proteins from RBP-MS with Lct −3′UTR RNA (*n* = 266). The top hit which is a possible RBP binding to the Lct-3′UTR region, is SNRNP70 (dark blue dot). Left side of the volcano plot is the enriched proteins on the Fluc RNA sequence (log_2_FC < 0). Gray dots are *p* value > 0.05 and cyan dots are *p* value < 0.05. Darker gray dots and darker cyan dots are known RBPs (Additional Files 2: Table S11). **e** smFISH images of the sIOs after *Snrnp70* knock-down by shRNA and control sIOs (scrambled shRNA). DAPI in blue and each GFP transcript dot in cyan or white/black. Scale bar 10 µm. Blue arrows indicate GFP-RNA granules in control sIOs

Previous studies have shown the importance of the 3′UTR for mRNA localization [39–42]. This sequence might thus be responsible for the localization of mRNAs which is independent of translation. To test this possibility, we first expressed chimeric mRNAs containing the GFP coding sequence fused to the 3′UTR region of either *Lct*,* Apob*, or *Net1* in sIOs, and visualized their localization pattern by smFISH (Fig. 4a). In all three cases, the 3′UTR was sufficient to replicate the localization pattern of their respective full-length mRNAs (Fig. 4b and Additional Files 1: Fig. S6a), indicating that, for these transcripts, the 3′UTR, and not the coding sequence or 5′UTR, is responsible for their asymmetric localization. We then hypothesized that these patterns of localization could be the result of RBP/mRNA interactions, as it has been proposed in other systems [39]. To test this hypothesis, we in vitro transcribed the 3′ UTR sequences of *Lct*, *Apob*, and *Net*, conjugated them to biotin and incubated them with sIOs lysates, followed by streptavidin pulldown and mass spectrometry (MS) (Fig. 4c). In each dataset, several RBPs were found to be enriched with respect to the control sequence (firefly luciferase, Fluc) (Additional Files 1: Fig. S6b,c,g). We found that UNK was highly enriched on the *Net1*−3′UTR (Additional Files 1: Fig. S6g). This corroborates our previous results describing UNK’s association with the *Net1*−3′UTR in neuronal cells [39], where it mediates its localization to neurites. Among other hits, isolation of the RPBs interacting with the 3′UTR of *Lct* revealed a marked enrichment for the U1 small nuclear ribonucleoprotein 70 K (SNRNP70), a member of the spliceosome complex (Fig. 4d). Despite being mainly localized in the nucleus, SNRNP70 has recently been proposed to also have a cytoplasmic role [43] and to localize to RNA granules within the axonal compartment, where it locally modulates the transcriptome [43]. To uncover the possible role of SNRNP70 as a mediator of mRNA localization in gut cells, we performed shRNA-mediated knock down (KD) in sIOs, where *Lct*−3′UTR localizes in apical granules (Fig. 4e). While *Actb*-mRNA showed no change in expression level and localization for both conditions (Additional Files 1: Fig. S6e and Additional Files 2: Table S8), SNRNP70 KD resulted in a sevenfold downregulation of *Lct*-mRNA abundance and a loss of *Lct*-mRNA foci structure in the apical side of epithelium (Additional Files 1: Fig. S6f and Additional Files 2: Table S8). This result demonstrates the role of SNRNP70 in mediating the localization and potentially expression of the *Lct*-mRNA in spite of the fact that our results cannot definitively determine whether granule formation is impaired directly, or as a consequence of transcript abundance. Moreover, this observation aligns well with the prior finding that SNRNP70 might influence the size of mRNA granules [43]. Together with our re-feeding experiments, these results illustrate a dynamic picture, in which *Lct*-mRNA localize within granular structures in a snRNP70-dependent manner. Taken together, our functional assays and extensive RNA maps shed light into the diversity of RNA localization patterns in intestinal epithelia and give account of the multiple mechanisms required for their formation.

### Spatial transcriptomics expands the sIO transcript localization atlas

**Fig. 5:**
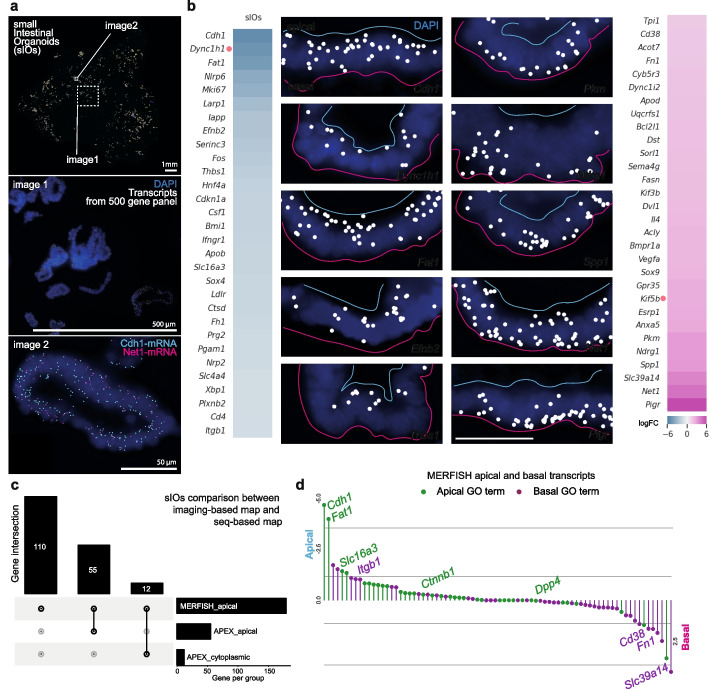
Imaging-based RNA map of small intestinal organoids (sIOs).** a** MERFISH images from sIOs sections, indicating transcripts (in rainbow or indicated colors) and cell nuclei in dark blue (Additional Files 2: Table S13). **b** Heatmaps of top 30 apically (left) and basally (right) enriched transcripts and representative images with log_2_ fold change (Additional Files 2: Table S14). Apical end of cells is marked with cyan line and the basal end with magenta. DAPI in blue. Scale bar 50 µm. Transcripts mentioned in the main text are indicated by a red dot. **c** UpSet plot. Comparison of the apical RNAs from imaging-based map (MERFISH) with seq-based map (APEX-seq) (Additional Files 2: Table S15). **d** List of transcripts categorized by apical GO terms (green) and basal GO terms (purple). Negative values stand for apical RNA localization and positive values for basal RNA localization

In order to further validate our biochemical approach and expand our analysis to other transcripts, we performed massively multiplexed error-robust single-molecule FISH imaging (MERFISH). This technique allows for the subcellular mapping of hundreds of RNA molecules in a single experiment [44]. We designed a MERFISH panel targeting 500 genes (Additional Files 2: Table S13). The panel included RNAs with unknown subcellular localization, targets identified in our APEX-seq dataset, as well as tissue marker genes to provide structural information of intestinal and surrounding tissues. Several thousand sIOs were embedded in the optimal cutting temperature compound (OCT) block, sectioned, and 388 of the 500 transcripts were detected (Fig. 5a). After calculating the log_2_ fold change (logFC) of RNA abundance between apical and basal regions (Additional Files 1: Fig. S7a and Methods), 66 transcripts were found to be more abundant in the apical side (logFC <  − 0.5) and 95 were more abundant in the basal side (logFC > 0.5). The top 30 most apical and basal transcripts are listed along with representative images (Fig. 5b). A paired Wilcoxon signed rank test was applied for each transcript, using each ROI as a replicate (*n* = 149). This analysis was made with volume normalized transcript counts, using Rstatix (v0.7.0). This demonstrates that, for many of the mRNA species analyzed, there is a significant localization bias (Additional Files 1: Fig. S7b and Additional Files 2: Table S16). When integrated with our APEX-seq data, we could assign 55 previously un-defined apically localizing transcripts and correct 12 transcripts which were previously categorized as cytoplasmically enriched to the apical region of the cells, including *Cdh1*-mRNA (Fig. 5b–d).

Interestingly, one of the most apically localized transcripts in our MERFISH dataset was *Dync1h1*, which encodes for a component of the cytoplasmic Dynein 1 complex, well-known in neurons for its role in retrograde axonal transport (toward the minus microtubule end) [45] (Fig. 5b). In contrast, *Kif5b-*mRNA was ranked among the top basal hits. KIF5b protein is a member of the kinesin family and is known to participate in anterograde axonal transport (toward the plus end of microtubules) [46] (Fig. 5b). In enterocytes, the minus end of the microtubules is oriented toward the apical side and the plus end toward the basal side [7, 47–51]. Some motor proteins have been found transporting their own mRNA [52]. Additionally, we classified the transcripts using the GO categories to test the extent to which the location of transcripts correlates with the predicted location of the protein. Fifty-five percent of the transcripts with apical GO terms were enriched in the apical region and 67% of the transcripts with basal GO terms were located in the basal region (Fig. 5d, Additional Files 2: Table S2 and Methods). These results suggest that transcript localization might correlate with where the encoded protein carries out its function.

### mRNA localization patterns change along the crypt-villus axis in small intestinal tissue

**Fig. 6:**
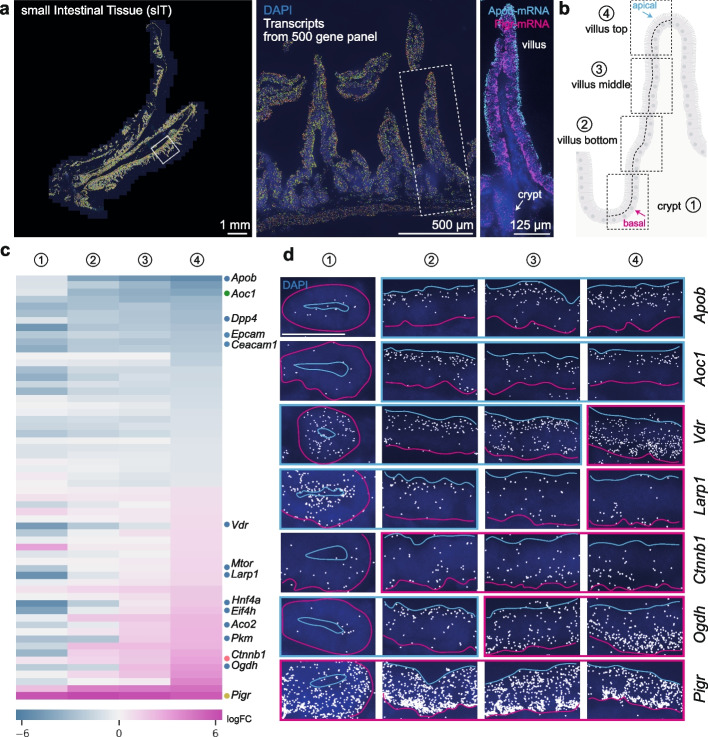
Imaging-based RNA map of small intestine tissue (sIT).** a** sIT section and transcripts (rainbow colored or indicated colors) detected by MERFISH. Dashed squares indicate location of zoomed in pictures. Intestinal crypt and villus are indicated. **b** Scheme indicating different zones for (1) crypt, (2) villus bottom, (3) middle, and (4) top and apical to basal, respectively. Figure created with BioRender.com. **c** Heatmap of top 30 apical and basal transcripts in 4 different zones with log_2_ fold change (Additional Files 2: Table S17). Genes are sorted according to localization in the villus top. Blue dot: nutrient sensing and digesting, green dot: apical membrane receptor and receptor binding, yellow dot: pathogen defense and red dot: junctional and cytoskeletal category. **d** Representative MERFISH images of indicated sIT zones and transcript species. Apical end of cells is marked with cyan line and the basal end with magenta. DAPI in blue. Border color of images indicates predominantly apical (cyan border) or basal (magenta border) localization patterns. Scale bar 25 µm

SIOs are excellent models to study intestinal biology [19]. However, the environment to which enterocytes are exposed in the intestine widely differs from that of culture. In intestinal tissue, nutrients and microbiota exist in the lumen and make contact to the apical side of epithelium. In order to expand subcellular transcriptome repertoire from in vitro system to in vivo, we performed MERFISH on a section of murine small intestine tissue (sIT). For comparison purposes, we used the same 500 probe panel as with the sIOs. Almost the totality of transcripts (498) were detected. Of these, 318 mRNAs show polarized patterns: 106 transcripts localizing to the apical and 212 to the basal side (see Methods) (Fig. 6a). The sIT presents long villi bearing discrete metabolic domains [52] and distinct crypts furrowed deeply in the extracellular matrix. Inspired by previous studies [16, 53], we divided the Gut in 4 different zones for further analysis: (1) crypt, (2) villus bottom, (3) villus middle, and (4) villus top (Fig. 6b). The different areas were differentiated using previously identified markers [16] (Additional Files 1: Fig. S8a,b).

We first analyzed the localization changes of the top 30 apically and basally localized transcripts of the villus top region with the villus middle, bottom, and crypts (Additional Files 1: Fig. S8c,d). Prominently, *Apob* (apically localized) and *Pigr* (basally localized) mRNAs maintained their strong localization bias through all regions (Fig. 6c,d). We found that 27/30 transcripts largely retained their apical localization pattern from crypt to villus top. Of the top 30 basally localized, only 9 kept their localization in all areas. Other transcripts, such as *Vdr*, *Mtor*, *Larp1*, *Eif4h*, *Aco2*, *Pkm*, and *Ogdh* localized to the apical region in the villus bottom and switched to basal localization in cells at the villus top (Fig. 6c). Interestingly, when the entire list of 318 polarized mRNA was considered, this transition was found in 120 RNAs, with 72/120 falling into the nutrient-related GO category. This result suggests that nutrient-related transcripts show dynamic RNA localization patterns based on different zones of the villus. Previously, we reported that mRNAs enriched in the apical end of epithelial cells have a twofold higher translation efficiency than basal transcripts [2]. We hypothesized that changes in localization of mRNAs along the length of the villi could regulate the translational output of certain mRNAs. We re-analyzed the data from Harnik et al. [53], where the authors collected epithelial cells from different zones within the villus and analyzed their protein composition using mass spectrometry. Interestingly, the protein levels of mTOR and Larp1 correlated with the changes in localization of their mRNA (decreasing from bottom to top of villi). However, whether this change in protein amount is a consequence of change in localization and subsequent changes in translational efficiency, or due to changes in expression levels of these mRNAs in the different zones along the length of the villus is to be determined. This insight still motivated us to dissect the relationship between RNA localization and protein levels on a broader scale. We analyzed the relationship between scaled RNA and protein expression in villus bottom and villus top separately. We could isolate 77 candidates present in both our MERFISH dataset and the mass-spec results from Harnik et al. We found no strong positive correlation between the two datasets (Pearson coefficient =  − 0.0995 in villus bottom and 0.0652 in villus top, Additional Files 2: Table S22) indicating that protein expression is governed not only by RNA itself but also by many other factors like translational control, protein stability, RNA stability, and RNA localization. This aligns with a previous study that reported a discordance of RNA and protein expression in the intestine [53].

Moreover, gut epithelial cells differentiate and mature along the crypt to villus-top axis, in a process that involves both tissue-intrinsic signals and environmental factors such as oxygen concentration or nutrient availability [15, 17]. We were able to identify localization changes of genes that play an important role in this maturation process. For instance, *Apob*-mRNA (whose product plays a role in transporting fat molecules) localizes apically throughout the intestine (Fig. 6c). However, the extent of this bias is altered along the crypt-villus axis, presenting a marked apical localization in the villus’ top and middle, where most of the absorption takes place [16, 53]. On the other hand, *Apob*-mRNA only exhibited a mild apical localization bias in crypts and the villus bottom. Another remarkable example is the Hepatocyte nuclear factor 4 (Hnf4a). We found its RNA to localize apically mainly in crypts (Fig. 6c). Hnf4a encodes for a master regulator of the brush border gene program, which is required for stabilization of enterocyte identity and maturation of the fetal intestine [54, 55]. This data suggests that RNA localization might be linked to the localization of the protein to facilitate physiological cell functions. One important consideration in this regard is that protein expression follows mRNA expression, leading to discordant expression along the cell maturation axis [53]. This phenomenon likely also affects RNA localization and its impact on protein translation, as reflected in our studies (this and Moor et al. [2]) and Harnik et al. [53] However, to establish a causative link further experimental work is required. In sum, this MERFISH dataset has newly defined apical and basal transcripts in sIT with high resolution, extending beyond the previously established maps (Additional Files 1: Fig. S8e).

### Zonated spatial atlas of large intestinal tissue

**Fig. 7:**
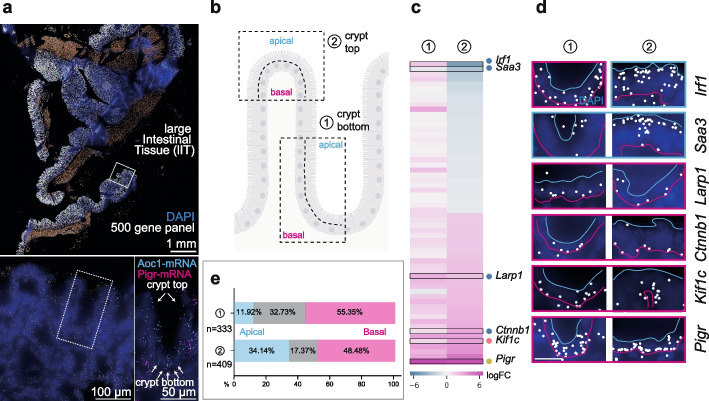
Imaging-based RNA map of large intestine tissue (lIT).** a** lIT section and transcripts (rainbow colored or indicated colors) detected by MERFISH. Dashed squares indicate location of zoomed in pictures. Crypt top and bottom are indicated. **b** Scheme of segmentation for (1) crypt bottom and (2) crypt top and apical to basal, respectively. Figure created with BioRender.com. **c** Heatmap of the top 30 apical and basal transcripts with log_2_ fold change (Additional Files 2: Table S20). Crypt top was taken as a reference region to select these genes. Blue dot: nutrient sensing and digesting, yellow dot: pathogen defense and red dot: junctional and cytoskeletal category. **d** Representative MERFISH images of indicated transcripts. The apical end of cells is marked with cyan line and the basal end with magenta. DAPI in blue. Scale bar 25 µm. **e** Bar plot of apically and basally localizing RNAs in percentage in two different zones (Additional Files 2: Table S21). (1) crypt bottom: apical 11.92%, basal 55.35%, non-localizing 32.73% and (2) crypt top: apical 34.14%, basal 48.48%, non-localizing 17.37%

The large intestine is a complex organ which is different in tissue architecture and function from the small intestine [56]. To better capture the diversity of RNA localization patterns in this tissue, we expanded our analysis to murine large intestinal tissue (lIT). We performed MERFISH using the same 500 gene panel as in previous cases (Fig. 7a). To analyze this sample, we divided the colon in two regions: (1) crypt bottom and (2) crypt top (Fig. 7b). Similar to the sIT data, we dissected crypt top and crypt bottom by different ROI assignments (Additional Files 1: Fig. S9a,b). We calculated the logFC between apical and basal sides for both crypt top and crypt bottom, and extracted the 30 most differential transcripts for both (Fig. 7c and Additional Files 1: Fig. S9c). We could find many transcripts localized in the basal side in lIT compared to sIT (Additional Files 1: Fig. S9d). Of note, lIT analyses revealed numerous RNAs shifting from apical localization at the crypt top to basal localization at the crypt bottom (Fig. 7c,d). Within our panel, there were less apically localizing transcripts in the crypt bottom (11.92%) than the crypt top (34.14%) (Fig. 7e). Among the transcripts, which showed apical localization in the crypt top but basal localization in the crypt bottom, we find that almost half of them (44/90 transcripts) belong to the nutrient sensing and digesting GO term category. There are innate differences in cell types and cell compositions between crypt bottom and crypt top, but also tissue functions between lIT and sIT, which might influence the RNA localization and its changes. Finally, we compared the RNA localization across different zones in sIT and lIT to explore overlapping transcripts between all zones and tissues (Additional Files 1: Fig. S9e). The highest overlap was found as basal transcripts between sIT top and sIT middle regions, with more than 200 transcripts (Additional Files 1: Fig. S9e). The second highest overlap was between the lIT basal and sIT apical crypt regions, showing about 200 transcripts to be localizing in different sides of the crypt in different tissues (Additional Files 1: Fig. S9e).

Together, our transcript localization analysis using proximity labeling and MERFISH in sIOs, sIT, and lIT provides an important resource to understand RNA localization. We believe that this atlas, generated using both chemical and imaging tools, represents a powerful platform for the generation of new hypotheses interrogating the physiological roles of asymmetric transcript localization in gut cells.

## Discussion

RNA localization plays fundamental roles in several contexts from embryo development to fundamental cell physiology including cell migration [3]. Yet, it is still unexplored across most cell types and tissues. The characterization of distinct subcellular transcript patterns is the first step toward the identification of the physiological roles of RNA localization. In this study, we charted subcellular RNA maps of intestinal epithelium using sequencing-based (APEX-seq) and imaging-based (MERFISH) methods. By leveraging the strengths of both tools, we defined the transcriptional profiles of the apical and basal regions of the intestinal epithelium, while also incorporating zonation information from the crypt bottom to the top. Our study highlights the necessity and potential of combining different high-throughput methods for the mapping of RNA. While APEX-seq uncovers subcellular RNAs unbiasedly, it requires introduction of the APEX peroxidase into cells. MERFISH enables observations limited to its targeted gene panel but can generate imaging data sets covering tissue or hundreds of sample sections. Furthermore, we included a thorough validation and quantification by smFISH (see for example, the case of *Cdh13*).

Generally, our data suggest that the localization of many transcripts might correlate with where their encoded protein exerts their function (Figs. 1, 2, and 5). Noticeably, we observed *Dync1h1* (Dynein Cytoplasmic 1 Heavy Chain 1) and *Kif5b* (Kinesin Family Member 5B) as one of the top apical and basal hits, respectively, in sIOs as well as sIT. Interestingly, the mRNA of motor protein KIF1C, a component of the Kinesin superfamily like Kif5b, interacts with its encoded protein, localizing asymmetrically in protruding end of melanoma cells. This specific RNA localization is governed by a short GA-rich element in the proximal 3′ UTR as well as several binding partners related to the cytoskeleton, integrin signaling, and vesicle transport, for instance Rab proteins. It is still unclear whether the localization of *Kif1c*-mRNA is required for proper KIF1C-mediated mRNA trafficking or cell migration in other cell types [52]. Our study suggests that this mechanism is not constrained to a specific cell type, but is present in other cell types and tissues. In those lines, it has been recently shown that mRNA localization in neurons and epithelial cells is partially correlated [7]. Co-transport of motor proteins and their RNA is likely not limited to *Kif1c* and could be a general principle for motor-protein mRNAs in cells, and, based on our data, it is possible that it also applies to Dynein complexes. Due to the vast family members in the field of motor proteins, the proteomics or immunofluorescence-based studies for individual members are to some degree limited. However, there are some findings using CLIP, RNA-seq, and fluorophore tagging assay [52], showing the high possibility of motor protein and its own RNA co-traveling and suggesting mRNA localization regulates its protein function. Therefore, our RNA chart can be a good resource to decipher the functional relationship between motor protein and its own mRNA.

While the localization of RNAs and their encoded proteins may correlate to a certain extent, our mechanistic studies, as well as the variety of localization patterns that each transcript can adopt in different cells along the gut, indicate that transcript localization also depends on other intracellular and environmental factors. In a previous report [2], we proposed that the translation machinery has a preferential apical distribution and that translation can be enhanced upon the re-feeding of fasted mice. Complementing these results, in this study, we have revealed that a subset of RNAs is affected by their translational state, others remain unaffected, while a subset of them partially change their localization or their aggregations state, highlighting the diversity of mechanisms by which this process might be regulated [4, 57].

Visualization of single mRNAs using smFISH also revealed the distribution of mRNAs within the cell, and we found that many apical mRNAs, especially transcripts coding for proteins related to digestion and nutrient sensing to localize within clusters. Whether these clusters represent translation factories as previously observed in case of Dync1h1 or are places of storage for mRNAs allowing the cell to respond to external stimuli is unclear. Further experiments, using live imaging of mRNAs using the SunTag system, can help distinguish between the two.

Interestingly, these structures are not always related to active mRNA translation, as they sometimes appeared (or were still formed) when interfering with translation. mRNA granules have been related to many processes [27, 28] (for instance, active translation, RNA degradation, or silencing), but their existence on epithelial cells is understudied. We proposed a factor related to granule formation in gut epithelium could be SNRNP70. We found this RBP to be critical for the formation of *Lct*-mRNA foci. SNRNP70 has been previously described to affect mRNA granule formation in neurons [43] as in line with our study we demonstrate total abrogation of *Lct*-mRNA granules.

## Conclusions

The intestinal epithelium has an intriguing architecture of protruding villus and furrowing of crypts which results in different concentration axes of nutrients and pathogens [16, 53]. This complex environment is accompanied by differential transcript expression in different gut areas [16, 53]. In this study, we have described another level of gene regulation, enabled by the wide diversity of the subcellular spatial patterns of transcripts. A major conclusion of our study is the realization that even within the same tissue, a single transcript can adopt radically different localization patterns. Systematical charting of the RNA localization by spatial transcriptomic methods serves as a rich resource showing that a large number of transcripts are asymmetrically localized. This study provides the molecular framework to further investigate the mechanisms of transcript localization and patterning and uncover their potential functions.

## Methods

### Mice

All experiments were performed on 9–14-week-old male and female mice. Chow and water were available ad libitum. All mice were in the C57BL/6 J background and maintained a 12-h light/12-h darkness schedule. Mice were housed and bred under specific pathogen-free conditions in accredited animal facilities. Mice were sacrificed by raising CO_2_ concentrations. All experimental procedures were performed in accordance with Swiss Federal regulations and approved by the Cantonal Veterinary Office.

### Mammalian cell culture

HEK293T cells (passages < 25) were cultured in a DMEM (Life Technologies, 31,966–021) supplemented with 10% fetal bovine serum (Corning, 35–079-CV), 1% PenStrep (Life Technologies, 15,140,122) at 37 °C under 5% CO_2_. Mycoplasma testing was performed before experiments.

### Generation of lentivirus of different APEX2 constructs

APEX2 fusion constructs in Supplementary Fig. 1a,d and 2a (in details, Additional Files 2: Table S23) were cloned into pLVX-IRES-ZsGreen1 (Takara-Clontech, 632,187), via InFusion (Takara, 638,949), between EcoRI and BamHI sites. For preparation of lentiviruses, HEK293T cells in T-150 plates were transfected at ∼60–70% confluency with the lentiviral vector pLVX containing the gene of interest (13.8 μg), the lentiviral packaging plasmids psPAX2 (10.4 μg; AddGene 12260) and pVSV-G (3.5 μg; AddGene 8454), and 50 μL of JetPrime reagent (Polyplus, 117–15) for 4 h and then replaced to the growth media. About 48 h after transfection the cell medium containing lentivirus was harvested and filtered through a 0.45-mm filter (Corning, 430,768). The harvested virus was concentrated by ultracentrifuge (1.5 h, 50,000* g*). Priorly 10 μM Y-inhibitor (StemCell Technologies, 72,304)-treated Organoid cells were then infected at ∼50% confluency. Transduction was facilitated with polybrene (final concentration is 8 μg/ml; Sigma-Aldrich, H9268). After 2 days, organoid medium was exchanged without Y-inhibitor [58]. To confirm transduction, all constructs also contained IRES-zsGreen within the same transcriptional unit (Additional Files 1: Fig. S2a,b).

### Small intestinal organoid

Cultures of the murine small intestinal were generated as previously described [19]. Briefly, crypts were isolated and purified from the murine small intestine, embedded in 50-μl matrigel drops (Corning, 356,231) and overlaid with 500 μl of complete IntestiCultTM Mouse Organoids Growth Medium Kit (StemCell Technologies, 06005) supplemented with 100 units/100 μg per mL of Penicillin–Streptomycin. The medium was replaced every 4 days. Established lines were passaged every 7 days by mechanical disruption with a bent P1000 pipette tip and were allowed to mature into large organoids for 4 to 5 days prior to every experiment.

For smFISH, cultures were harvested by aspiration of the medium and kept on ice for 30 min with organoid harvesting solution followed by a brief PBS wash, and collected by scraping the matrigel roughly with a truncated P1000 tip into Eppendorf tubes. Then, samples were centrifuged at 300* g* for 2 min and the matrigel was aspirated, followed by fixation at 4 °C with 4% PFA (Santa Cruz Biotechnology, SC-281692) for 45 min, and permeabilization at 4 °C with 70% EtOH for 1.5 h before embedding in OCT (Tissue-Tek, 4583) [24].

For starvation and re-feeding experiment, sIOs were passaged for 4 days priorly and the growth media is exchanged to Earle’s balanced salt solution (EBSS, Life Technologies, 14,155,063) for 6 h [29]. Then for staving, EBSS was exchanged with EBSS for 2 h and for re-feeding, EBSS was exchanged with the fresh growth media for 2 h.

For the chemical perturbation experiment, sIOs were incubated with a fresh growth medium containing 20 or 200 μM puromycin (InvivoGen, ant-pr-1) for 30 min, 100 μg/ml cycloheximide (Sigma-Aldrich, C7698) for 30 min, 3 μg/ml harringtonine (Sigma-Aldrich, SML1091) for 60 min, 100 μM sodium arsenite solution (Sigma-Aldrich, 1,062,771,000) for 60 min or DMSO (Life Technologies, 85,190) for 30 min at 37 °C under 5% CO_2_, and then immediately processed for fixation.

### APEX labeling in living cells

Four days after plating organoids stably expressing the corresponding APEX2 fusion construct, organoids were harvested using cold PBS without damaging the organoid. The general APEX-seq protocol [4, 18] is applied but slight modifications are made to adapt to the organoid system. APEX labeling was initiated by changing the growth medium to fresh growth medium containing 500 μM biotin-phenol (Iris Biotech GMBH, LS-3500). This was incubated at 37 °C under 5% CO_2_ for 30 min. H_2_O_2_ (Sigma-Aldrich, H1009) was then added to each well to a final concentration of 1 mM, and the plate gently agitated for 1 min [4, 5]. The reaction was quenched by replacing the medium with an equal volume of 5 mM Trolox (Sigma-Aldrich, 238,813), 10 mM sodium ascorbate (Sigma-Aldrich, 11,140) and 10 mM sodium azide (Brunschwig, ACR44781-0250) in Dulbecco’s phosphate-buffered saline (DPBS, Life Technologies, 14,190,144). Cells were washed with DPBS containing 5 mM Trolox and 10 mM sodium ascorbate three times before proceeding to imaging, RT-qPCR, or RNA-seq experiments. The unlabeled controls were processed identically, except that the H_2_O_2_ addition step was omitted.

### Immunofluorescence staining and fluorescence microscopy

Organoids, grown in a 8-well chamber, were fixed with 4% paraformaldehyde in PBS at room temperature for 20 min. Organoids were then washed with 3% BSA in PBS three times and permeabilized with 0.5% Triton X-100 (Sigma-Aldrich, X100) in PBS at room temperature for 20 min. Organoids were washed again three times with 3% BSA in PBS and blocked for 1 h with 1% BSA, 0.2% Triton X-100, 0.05% Tween (“blocking buffer”) at room temperature. Organoids were then incubated with primary antibodies (mouse anti-V5 antibody 1:200 dilution, Life Technologies, R960-25; rabbit anti-EZR 1:200 dilution, abcam, erp233353; rabbit anti-TOM20 antibody 1:200 dilution, abcam, ab221292) in blocking buffer overnight at 4 °C. After washing three times with PBS, organoids were incubated with secondary antibodies (goat anti-mouse AlexaFluor488, 1:400 dilution, Life Technologies, 11,001; goat anti-rabbit AlexaFluor594, 1:400 dilution, Life Technologies, 11,012; streptavidin-AlexaFluor647, 1:400 dilution, Life Technologies, S32357) in blocking buffer for 1 h. Organoids were then washed three times with PBS, stained with DAPI (1 µg/mL; Life Technologies, 62,247) for 30 min, again washed three times with PBS and imaged [35]. Fluorescence confocal microscopy was performed with a Leica SP8/Falcon SP8 microscope with × 63 oil immersion objectives, outfitted with a Yokogawa spinning disk confocal head, Cascade II:512 camera, a Quad-band notch dichroic mirror (405/488/568/647), and 405 (diode), 491 (DPSS), 561 (DPSS), and 640 nm (diode) lasers (all 50 mW). CFP (405 laser excitation, 445/40 emission), Venus/Alexa Fluor488 (491 laser excitation, 528/38 emission), AlexaFluor568 (561 laser excitation, 617/73 emission), and AlexaFluor647 (640 laser excitation, 700/75 emission) and differential interference contrast (DIC) images were acquired through a × 63 oil-immersion lens. Acquisition times ranged from 100 to 2000 ms.

### RNA extraction for RT-qPCR or RNA-seq

To labeled and unlabeled (controls) organoids in 6-well plates, we added ∼1 ml DPBS containing 5 mM Trolox and 10 mM sodium ascorbate, as well as ∼4 μl RNase inhibitor (Lucigen, 30,281–2). The organoids were spun down at 300* g* for 2 min, transferred to 1-mL Eppendorf tubes, and spun at ∼400* g* for 4 min to pellet cells. The supernatant was removed, and the RNA was extracted from cells using the RNeasy plus mini kit (QIAGEN) following the manufacturer’s protocol, including adding β-mercaptoethanol to the lysis buffer. The cells were sent through the genomic DNA (gDNA) eliminator column supplied with the kit. A modification to the protocol was replacing the RW1 buffer with RWT buffer (QIAGEN) for washing. The extracted RNA was eluted into RNase-free water, and RNA integrity was checked using the Agilent bioanalyzer 2100 using the RNA pico assay. Only RNA with a RIN (RNA integrity number) > 8.5 was used for subsequent experiments. RNAs shorter than 100 nt were not efficiently recovered. RNA concentrations were determined using the Nanodrop (Life Technologies).

### APEX labeling streptavidin dot blot experiment

RNA from labeled organoids was treated with Turbo DNase (Life Technologies, AM2235) at 37 °C for 30 min, followed by purification using the RNA clean and concentrator − 5 kit (Zymo Research). ∼500 ng of purified RNA was blotted on the Amersham Protran 0.45 nitrocellulose (NC) membrane, and the membrane allowed to sit for at least 15 min to allow liquid to dry. The RNA was Crosslinked to the membrane two times using 2500 μJ energy (254 nm wavelength, UV Stratalinker 2400) (Spitale et al., 2015). The membrane was then wet with ∼5 mL PBST (PBS-TWEEN 20), followed by incubation with 15-mL PBST containing 1 μL LI-COR Streptavidin IRDye green 800CW (LiCor, 925–32,232). The membrane was washed with PBS and imaged on the LI-COR Odyssey CLX. For the RNase digestion, we treated the RNA with RNase cocktail enzyme mix (Life Technologies, AM2286) for 30 min at room temperature (RT), followed by purification using the RNA clean and concentrator kit.

All RNA experiments were carried out using standard protocols to minimize and eliminate RNase contamination. These included using a dedicated work area for RNA, using filtered pipette tips, wiping all surfaces with RNase Zap (Life Technologies, AM9780), using certified RNase-free buffers and reagents. When appropriate, ∼1–2 μl of Ribolock RNase inhibitor (Life Technologies, EO0387) was added per 100–200 μL of buffer/solution.

### Enrichment of biotinylated RNA

To enrich biotinylated RNAs, we used Pierce streptavidin magnetic beads (Life Technologies, 88,816), using 4–5 μl beads per 10 μg of RNA. The beads were washed 3 times in B&W buffer (5 mM Tris–HCl pH = 7.5, Life Technologies, 15,567,027; 0.5 mM EDTA, Life Technologies, AM9260G; 1 M NaCl, Life Technologies, AM9760G; 0.1% TWEEN 20, Sigma-Aldrich P9416), followed by 2 times in Solution A (0.1 M NaOH, VWR SS055 and 0.05 M NaCl) and 1 time in Solution B (0.1 M NaCl). The beads were then suspended in ∼100 μL 0.1 M NaCl and incubated with ∼125 μl RNA (diluted in water) on a rotator for 2 h at 4 °C. The beads were then placed on a magnet and the supernatant discarded. Beads were washed 3 times in B&W buffer and RT was made directly on the beads using previously published msSCRB-seq protocol [59].

### APEX RT-qPCR experiments (MITO)

To test for APEX RNA enrichment, we designed primers against positive, Mtnd1 and negative, Actb. The sequences of the primers (purchased from IDT) are listed in Additional Files 2: Table S24. For the RT-qPCR experiments, the enriched MITO-APEX2 RNA was first reverse transcribed following the Superscript III reverse transcriptase (Life Technologies, 12,574,026) protocol using random hexamers as primers. The resulting cDNA was then testing using qPCR using the primers above in 2X SYBR Green PCR Master Mix (Life Technologies, c14512), with data generated on QuantStudio 3 (Life Technologies). For each RNA, we calculated the ratio of RNA recovered in the labeled target relative to unlabeled controls. We then calculated enrichment as recovery of positives relative to negatives, correcting for primer efficiency (> 85% for all primers).

### APEX-seq library preparation

RNA-seq libraries were prepared from extracted and enriched RNA using the Illumina Nextera XT DNA library preparation kit (Illumina, TG-131–2001). The prepared libraries were indexed and their quality was tested with a TapeStation 4200 system (Agilent Technologies) using D1000 High Sensitivity Screen regents and tapes (Agilent #5067–5585, #5067–5584). As a second quality control libraries were first sequenced at shallow depth on a Illumina NovaSeq 6000 system using NovaSeq SP Reagent Kits (1000 cycles) v1.5 according to the manufacturer’s instructions. Prepared libraries were sequenced at ∼40 million paired (2 × 75) reads per library.

### Bulk RNA-sequencing analysis

Reads were demultiplexed with Bcl2fastq (v2.20.0.422, Illumina) and quality-checked with FastQC (v0.11.9, https://www.bioinformatics.babraham.ac.uk/projects/fastqc/). Any adapters found in the FastQC results were trimmed with cutadapt [60] (v4.0). STAR aligner [61] (v2.7.9a) was used to align the reads to Mus musculus reference genome GRCm38. The HTSeq count module [62] (v1.99.2) was used with the default mode to count the reads mapped to the genes. MultiQC [63] (v1.9) was used to generate an overall quality report after the alignment was done.

The counts matrix was imported into R 4.1.0. As a quality control, total mapped reads, uniquely mapped read percentage, percentage of reads that were mapped on genes and total gene counts with 3, 5, 10, 20, and 50 read thresholds (i.e., how many genes are remaining with different thresholds) were checked per sample and visualized in bar plots. These plots were examined visually and the samples that showed a steep decrease in these QC metrics were filtered out due to poor quality. Samples that were prepared on different dates were corrected for batch effect using ComBat-seq [64]. Genes with less than 5 counts were filtered out before a TMM normalization was applied using edgeR library [65] (v3.36). After the normalization, differential expression (DE) analysis was performed using edgeR and results were visualized using ggplot2 [66] (v3.3.6) and ComplexHeatmap [67] (v2.10). *Z*-scores for the heatmaps were calculated for each gene by subtracting the mean expression value of the gene across all samples from the gene expression value and dividing it to the standard deviation of the gene expression across all samples. All of the reported fold changes (logFC) are based on log_2_.

From the results of the DE analysis, gene set enrichment analysis was performed using clusterprofiler library [68] (v4.2.2). A list of ranks was calculated with the formula of -log(*x*)/sign(*y*) where *x* is the *p* value and *y* is the log_2_ fold change of the DE results. This list was used in gene set enrichment of Gene Ontology (GO) terms. Furthermore, a list of GO terms was curated in relation to known functions of small intestines (Additional Files 2: Table S2). These GO terms were extracted from the GO.db library (v3.14) and the gene identifiers were matched to highlight which genes belong to which GO terms. In the curated list of GO terms, genes that were known to be long non-coding RNAs and pseudo genes were excluded from the total count of genes in each category, as was reported in Fig. 1d. Curated list of GO terms are referred to as GO categories and in the case a gene is part of multiple GO categories, the highest priority GO category was assigned to the gene in the figures. Two different prioritizations were considered based on the analysis. In the sequencing results, GO category priority was as follows: Nutrient sensing & digesting > Apical membrane receptor & receptor binding > Pathogen defense > Junctional & cytoskeletal > Other. For imaging-based analyses GO category priority was as follows: Basal membrane receptor & receptor binding > Apical membrane receptor & receptor binding > Nutrient sensing & digesting > Pathogen defense > Junctional & cytoskeletal > Other. All GO categories belonging to a gene were always added in additional files of the respective analysis as well.

Fifteen thousand three hundred thirty genes remained for the DE analysis between DPP4 and GFP tagged samples. Two different log_2_ fold change (logFC) thresholds were checked to determine how many genes were considered to be significantly different between the samples. With the thresholds of |logFC|≥ 1, 1211 and 1128 genes were significantly enriched in DPP4 and GFP samples, respectively. Lowest enrichment in statistically significant genes within the same threshold were 1.16 and 1.19 for DPP4 and GFP samples, respectively. The threshold of |logFC|≥ 2 further decreased the number of genes to 975 and 870 in DPP4 and GFP samples, respectively. In all cases, FDR *p* value was set to ≤ 0.05.

### Single molecule fluorescence in situ hybridization

smFISH staining was performed according to a previously published protocol [24, 40] with minor adaptations. Briefly, an 8-μm thick organoid section was placed on poly-L-lysine (Sigma-Aldrich, P8920) pre-coated cover glass. The sample was washed with cold PBS and fixed in 4% paraformaldehyde (Santa Cruz Biotechnology, sc-281692) in PBS for 10 min and subsequently washed two times with cold PBS. Fixed samples were permeabilized with 70% ethanol for at least 1 h or maintained overnight at 4 °C. The permeabilized samples were washed once with 2 × SSC (Life Technologies, J60561.AP), smFISH wash buffer containing 2 × SSC and 15% formamide (Life Technologies, AM9342) in nuclease-free water (Life Technologies, AM9932) for 5 min each at RT. The sample was hybridized with 1:50 ratio (5 μl smFISH probe:150 μl hybridization mix) overnight in 30 °C in a wet chamber. Probe libraries were designed using the Stellaris FISH Probe Designer (Biosearch Technologies, Inc., Petaluma, CA) and covalently coupled to Cy5 or Alexa 594 [24]. Hybridization mix was carefully removed and the sample was washed once with 2 × SSC at 37 °C for 30 min each in the dark. Samples were stained with DAPI (10 μg/ml in wash buffer; Life Technologies, D1306) for 30 min at 37 °C in the dark. DAPI solution was aspirated and samples were washed once with smFISH wash buffer for 5 min. Samples on cover glass were gently mounted upside-down with a small drop of ProLong™ Gold (Life Technologies, P36930). smFISH imaging was performed on a Leica THUNDER Imager 3D Cell Imaging system. × 100 NA = 1.4 oil immersion objective lens was used.

### Spot detection

3D images were processed to 2D maximum intensity projections using ImageJ which were used for further analysis. Spot detection was carried out using FISH-Quant [69] in python. Threshold for each image was determined using the automatic thresholding option in which there is an evidential drop resulting from the fast decreasing false positive detections (low intensity noise) and the slowly decreasing true positives. Apical and basal allocation of mRNAs was done manually. ROIs corresponding to the apical and basal mRNAs were drawn using the polygon function napari and spots lying within these regions were assigned as “apical” and “basal” respectively. The number of spots was normalized to the total area of the region to account for bias in cell size in the apical and basal regions. For the gradient spot detection, the degree of apical and basal quantification was carried out using napari as follows. Polygon ROIs corresponding to cells were drawn manually to demarcate cellular boundaries. A centerline was then drawn using the center of the nucleus as a reference. Spot detection was carried out with FISH-Quant as described above. The perpendicular distance between each spot and the center line was then calculated and normalized with the maximum width of the ROI.

### MERFISH

For MERFISH, standard procedures for fixed frozen tissue preparation as recommended by Vizgen were followed. In brief, organoids, small intestinal tissue, and large intestinal tissue from murine were fixed with freshly prepared 4% PFA in × 1 PBS for 16–24 h at 4 °C. For this study, MERFISH was performed for a single organoid, small and large intestinal tissue each. Next, 15% sucrose in 1 × PBS was applied for 6–12 h and the samples were transferred to 30% sucrose in 1 × PBS at 4 °C. All the liquid was removed and pre-chilled OCT was applied to freeze tissue with dry ice. For organoids, 70% ethanol was applied and all the liquid was removed by drying it (approximately 5 min). The samples were stored at − 80 °C. The fixed frozen sample was sectioned to 10-µm thickness on cryostats (Leica 3050 S). The section was mounted onto a functionalized 20-mm coverslip pre-treated with poly D Lysine. After placing on the coverslips for 5 min at − 20 °C, the samples were placed in 60-mm petri dish, washed in 1 × PBS for 5 min 3 times, washed in 70% ethanol and then stored in 70% ethanol at 4 °C. They were stored for at least 1 day and no more than 1 month before proceeding. Autofluorescence quenching for small and large intestinal tissue was made by using MERSCOPES photobleaching feature. The parafilm-sealed petri dish bearing the samples on coverslip was placed in the MERSCOPE photobleacher and left at room temperature for at least 3 h. The rest of the procedures for hybridization with a 50-μl droplet of MERSCOPE Gene Panel Mix (VA00255), gel embedding, and imaging were followed as provided by the manufacturer. After the imaging acquisition, segmentation was done manually using MERSCOPE Vizualizer (more than 150 ROIs were drawn in each dataset) and data was extracted for further analysis.

### MERFISH data analysis

ROIs that were drawn in MERSCOPE Visualizer were analyzed for the transcript counts in each region. Each ROI consisted of a main region that was split into two sides: apical and basal. To ascertain the localization of each transcript, spot counts normalized by the volume of the ROI were used. Following steps were done to create a normalized log_2_ fold change value that show localization information for each gene:

1. Difference between total number of basal counts per gene and apical counts per gene was calculated
2. Resulting value was divided by the count range of each region and was multiplied by 100
3. Lastly, the formula log_2_(|*x*|+ 1) if *x* >  = 0 and -log_2_(|*x*|+ 1) if *x* < 0, where *x* is the resulting value from previous step, was used to show log_2_ fold change with the information of enriched side of the region (negative values show apical enrichment, positive values show basal enrichment)

Resulting fold changes were considered to show higher basal abundance for > 0.5 and apical for <  − 0.5. Additionally, a paired Wilcoxon signed rank test was applied for each transcript, using each ROI as a replicate. This was done with volume normalized transcript counts, using Rstatix (v0.7.0) [70] in R.

Calculation of total number of spots per region was done similarly. For each region, the sum was calculated from volume normalized spot counts. Additionally, these sums were scaled as described in the second step above and log_2_ transformed to shrink the variance.

### UpSet plot

The UpSet plots, as introduced by Lex et al. [71], were generated using the “UpSetPlot” library in Python. We used UpSet plots to visualize the intersections among gene lists predominantly expressed in the apical and basal regions of the samples. The sorting of subsets was performed in descending order based on the intersection size.

### In vitro transcription and pull-down experiments for RBP-MS

Ten microliters (∼20–35 ng/µl) of cleaned-up PCR amplicon (primer sequences are provided in the Additional Files 2: Table S25, used with screening pooled library) was used as template of the in vitro transcription (HiScribe™ T7 Quick High Yield RNA Synthesis Kit; New England Biolabs, E2050S), performed at 37 °C for 16 h, followed by DNAseI treatment (37 °C for 15 min). IVT RNAs were then cleaned up and concentrated (DNA Clean & Concentrator-5; Zymo Research, D4013).

3′-Desthiobiotin labeling was carried out following the manufacturers’ guidelines of Pierce™ RNA 3′ End Desthiobiotinylation (Life Technologies, 20,163). Briefly, ∼115 pmol of each RNA were first subjected to fast denaturation in the presence of 25% v/v DMSO (85 °C for 4') to relax second structures, and subsequently labeled at 16 °C for 16 h. RNA-binding proteins were isolated by the means of Pierce™ Magnetic RNA–Protein Pull-Down Kit (Life Technologies, 20,164). Briefly, 3′-desthiobiotin labeled RNAs were incubated with magnetic streptavidin-coated beads (50 µl of slurry)/each RNA probe) for 30′ at room temperature, under agitation (600 RPM in a ThermoMixer, Eppendorf) [39]. Two hundred micrograms of cell lysates (in Pierce IP lysis buffer; Life Technologies, 87,787), derived from 4-day-old sIOs, were then incubated with 3′-desthiobiotinilated-RNA/streptavidin beads at 4 °C for 1 h under agitation (600 RPM). Final elution was performed in 50 µl/pull-down. Twenty microliters of each eluate was then analyzed by M/S. Incubation times are as follows: 30 min at RT, 600 RPM agitation for the binding of the labeled RNA to the beads; 1 h at 4 °C, 600 RPM agitation for the RBPs to the RNA and 15 min at 37 °C, 600 RPM agitation for the elution.

### Mass spectrometry

The pulldown samples were subjected to trichloroacetic (TCA) precipitation: 20 μl of each sample + 80 μl of H2O + 100 μl of 10% TCA (5% TCA end concentration). The resulting protein pellets were washed twice with cold acetone, dried, and dissolved as follows: 45 μl of 10 mM Tris/2 mM CaCl_2_, pH 8.2 buffer; 5 μl trypsin (100 ng/μl in 10 mM HCl); 0.3 μl trypsin Tris 1 M, pH 8.2 to adjusted to pH 8. The samples were then processed with microwave-assisted digestion (60 °C, 30 min) and dried. The dried digested samples were dissolved in 20 μl ddH_2_O + 0.1% formic acid; transferred to the autosampler vials for liquid chromatography-mass spectrometry analysis (LC–MS/MS); 2 μl were injected on a nanoAcquity UPLC coupled to a Q-Exactive mass spectrometer (Life Technologies).

The protein identification and quantification were performed using MaxQuant v1.6.2.3, and the data were searched against the Swissprot mouse database.

### Proteomic analysis

Quantified proteins were analyzed in R 4.1.0. Unique MaxLFQ values, as described by Cox et al., 2014, were used for each protein to test abundance differences between samples [59]. Proteins that were not quantified in more than 75% of the samples were filtered out from the analysis. DESeq2 [72] (v1.34) library was used to test the abundance differences. For the visualization of the results, ggplot2 (v3.3.6) was used. To highlight RNA-binding proteins (RBPs), biomaRt (2.50.3) was used to retrieve the description of the proteins from uniprot identifiers. Any description that contained “RNA binding” for a protein was highlighted.

### RBP knockdowns

Multiple shRNAs (2 shRNAs/protein) targeting mouse SNRNP70 were obtained from IDT. Primer information is here: Additional Files 2: Table S26. pLVX-shRNA vector was transfected to HEKT cells to obtain lentivirus. The lentivirus was applied to the sIOs and after checking EGFP expression level, total RNAs were collected for each sample, for instance the shRNA applied sIOs and the control shRNA applied sIOs, respectively. The efficiency of the knockdown was confirmed by qRT-PCR (Snrnp70 and EGFP). The RNA was collected and purified using Macherey Nagel XS RNA kit (Macherey Nagel, 740,902.50) followed by DNase I treatment for 15 min at RT. cDNA from 500 ng of purified RNA from each fraction was synthesized using RNA to cDNA EcoDry Premix (Takara, 639,543). qPCR was carried out using PowerTrack SYBR Mix (Life Technologies, c14512), 10 μl of 1:2 diluted cDNA, and primers (IDT, see sequences below) against the coding sequence of endogenous GAPDH (control) and Snrnp70 mRNAs. Reactions were carried out using the QuantStudio Real-Time PCR Systems with the following conditions: polymerase activation at 95 °C for 3 min and 40 cycles of 95 °C for 10 s, and 60 °C for 30 s. Finally, a melting curve was performed by incubating samples at 65 °C for 15 s followed by a temperature gradient increase at 0.5 °C/s to 95 °C. Each sample was measured with three technical replicates. To ensure no contamination, no template controls were performed. The knockdown efficiency was calculated using the ΔΔCt method with Snrnp70, EGFP and Gapdh primers (Additional Files 2: Table S27). MIQE guidelines were followed for all qPCR experiments.

## Supplementary Information

Additional files 1: Supplementary Figs. 1–9. Fig. S1: Proof of concept of APEX-seq in sIOs. a, Left: Scheme of the COX4 construct (also known as MITO-V5-APEX2) used as a bait to capture RNAs localizing inside of mitochondria. Right: Representative immunofluorescence images of COX4-V5-APEX2 expressing organoids. Scale bar 20 µm. b, qPCR results for the mitochondrial RNA, *Mt-nd1*. Results of ratio paired t test are indicated. Each dot represents one sample. Median indicated as the black bar. c, Correlation plot between Mitochondria samples with and without H_2_O_2_. A regression line is fit to log_2_ transformed counts per million (CPM) normalized counts to show the expression trend of the samples. Highly expressed mitochondrial genes are highlighted with dark blue color and labeled with their names (Additional Files 2: Table S4). d, Left: Scheme of the ACTB construct used as bait to capture RNAs localizing to the cytoplasm. Right: Representative immunofluorescence images of ACTB-V5-APEX2 expressing organoids. Scale bar 20 µm. e, GSEA results of ACTB enriched transcriptome from differential expression analysis using GO terms and normalized enrichment score (NES) (Additional Files 2: Table S5,6). Fig. S2: Quality control of APEX sequencing. a, Schematic representation of the constructs of DPP4 (apical bait) and GFP (cytoplasmic bait) attached to APEX2 machinery. b, Representative fluorescence of sIOs 2 days after lentiviral transduction. Scale bar 200 µm, upper panel and 20 µm, lower panel. c, Representative immunofluorescence images of sIOs stained with Streptavidin-A647 indicating biotinylated molecules in gray. Location of insets indicated with white Dashed boxes. DAPI in blue. Scale bar 20 µm and 5 µm for inset. d, The RNA integrity number (RIN) is shown for different samples. High RNA quality is insured both plus and minus H_2_O_2_ application. e, Dot blot with Streptavidin-A680. The biotinylated RNA is detected via staining with streptavidin. f, Representative correlation plots between two replicates for DPP4 and GFP samples. Each dot shows a gene and axes show normalized gene expression value for the respective sample. *R* value indicates Pearson correlation coefficient and *p* value shows the significance of calculated correlation. g, Principal component analysis (PCA) plot of samples that passed quality control (left) and percentage of explained variance by each principal component (right). h, Comparison with LCM-seq [2] (Additional Files 2: Table S6). Fig. S3: smFISH image analysis—spot detection. a, Spot detection in smFISH images. Each red dot is a smFISH dot. b, Example of segmentation of apical and basal region of cells shown on the *Actb* image. Lower row indicated insets in the upper row. c, smFISH image of *Cdh13*-mRNA. DAPI in blue and each transcript dot in white/black. Scale bar 20 µm. d, For gradient spot detection, the relative distance of each spot to the center line was considered for both apical and basal spots. Images on the right illustrate examples of spot detection and center line placement. The analysis on the right shows the spot distribution pattern relative to the total cell length for *Apob*-, *Net1*- and *Cdh13*-mRNA. Each dot indicates an individual transcript. Median indicated in the black bar (Additional Files 2: Table S9). Fig. S4: smFISH image analysis—foci detection. a, Diameter of RNA foci of indicated transcripts in µm (Additional Files 2: Table S10). The color of each bar plot represents their localization determined by APEX-seq (gray = unbiased, cyan = apical, magenta = basal). b, Representative smFISH images of starved and re-fed sIOs. DAPI in blue and each transcript dot in white/black. Scale bar 10 µm. White arrows indicate *Lct* granules in re-fed organoids. c, Quantification on smFISH dots normalized per area (dots/µm^2^): *Lct*-mRNA and *Actb*-mRNA. Unpaired t test with Welch’s correction applied (two-tailed *p* val *** < 0.0001, others ns > 0.05). d, Diameters of foci in µm. The number of RNA granules with > 0.5 μm diameter is indicated for each condition. Unpaired t test with Welch’s correction applied (two-tailed *p* val **** < 0.00001, ** < 0.001, ns > 0.05). Fig. S5: smFISH image analysis – perturbation on translation. a, Representative smFISH images of chemically perturbed sIOs. DAPI in blue and each transcript dot in white/black. Scale bar 10 µm. b, Diameters of foci in µm (Additional Files 2: Table S10). Unpaired t test with Welch’s correction applied (two-tailed *p* val *** < 0.0001, ** < 0.001, ns > 0.05). Gray shows non polarized pattern, cyan for apical and magenta for basal. c, Quantification on smFISH dots normalized per area: *Enpep*-, *Lct*- and *Actb*-mRNA (Additional Files 2: Table S8). Unpaired t test with Welch’s correction applied (two-tailed *p* val ** < 0.001, others ns > 0.05). Gray shows non polarized pattern, cyan for apical and magenta for basal. Fig. S6: 3’UTRs carry important signals for RBP binding and for RNA localization. a, Quantification of GFP transcript spots in smFISH (Additional Files 2: Table S8). An unpaired t test with Welch’s correction is applied. *** *p* val < 0.0001, ** *p* val < 0.001. b, PCA plot of all samples from the RBP-MS data. c, Volcano plot of the RBP-MS data. The left side is RBPs enriched in the *Fluc*-mRNA binding fraction and the right side is Lct-3’UTR enriched RBPs (Additional Files 2: Table S11). d, qPCR data showing *Snrnp70* is downregulated in the SNRNP70 shRNA applied sIOs compared to scrambled shRNA applied sIOs, while *eGFP* is not downregulated. Each dot represents technical replicates. Results of ratio paired t test are indicated. * *p* val < 0.01. Black line indicates the median. Relative gene expression normalized to GAPDH (2 − ΔΔCT). e, Actb-mRNA smFISH images of the sIOs after *Snrnp70* knock-down by shRNA and control sIOs (scrambled shRNA). DAPI in blue and each Actb transcript dot in white/black. Scale bar 10 µm. f, Left: Ratio spot density of apical to overall detected in smFISH for GFP transcripts. A Welch's t test is applied. * *p* val < 0.01 (Additional Files 2: Table S8). Right: Quantification on Lct-3’UTR smFISH dot diameter. Unpaired t test with Welch’s correction applied (two-tailed *p* val **** < 0.0001) (Additional Files 2: Table S10). g, Volcano plot of the RBP-MS data. The left side is Fluc enriched RBPs and the right side is Net1-3’UTR enriched RBPs (Additional Files 2: Table S12). Fig. S7: MERFISH image of a sIO section. a, Example of segmentation of apical and basal region using MERSCOPE Vizualizer. Transcripts detected from the 500 gene panel depicted in rainbow colors. PolyT (green) masks the whole cell contour: apical ROIs (toward the inner lumen) in light blue and basal ROIs in purple. Scale bar 100 µm. b, Normalized expression in apical and basal regions reveals apically and basally localizing RNAs in sIOs. The boxes show the quartiles of the data points with each dot representing one ROI. Paired Wilcoxon signed rank test is applied (Additional Files 2: Table S16). **** *p* val < 0.00001, *** *p* val < 0.0001, ** *p* val < 0.001 and * *p* val < 0.01. Fig. S8: MERFISH analysis of sIT section. a, Example of segmentation of apical and basal regions in (1) crypt, (2) villus bottom, (3) middle and (4) top, respectively. Numbers in image on the left indicate different regions for segmentation as well as apical (a) and basal (b) subregions. Transcripts for *Ada, Nlpr*6 or *stem cell markers* (see subfigure b) are depicted (Additional Files 2: Table S18). b, Localization of select transcripts confirms expected expression patterns. For example, stem cell markers (i.e., *Olfm4, Sox9, Sox4, Igfbp4, Myc, Ung* and *Cd44* in black) are highly localized in crypts (image1). *Nlrp6*-mRNA in yellow is gradually localizing from villus bottom (image2) to villus top (image3) and *Ada*-mRNA is exclusively localizing in villus top in red (villus top marker, image3). c, Heatmap for top 30 apical and basal transcripts in different zones of sIT with log_2_ fold change. Villus top was taken as a reference region to select top-ranked genes (Additional Files 2: Table S17). d, The top 30 apical and top 30 basal transcripts of each zone of sIT with log_2_ fold change (Additional Files 2: Table S17). e, UpSet plot to compare different zones in sIT and sIOs and apical to basal, respectively (Additional Files 2: Table S19). Fig. S9: MERFISH analysis of lIT section. a, Example of segmentation of apical and basal regions in (1) crypt bottom and (2) crypt top, respectively. Crypt top and crypt bottom markers (see subfigure b) are indicated in insets. b, Stem cell marker transcripts (for example, *Sox9, Mki67,* and *Cd44* in light gray) are localized in the crypt bottom (image1). Crypt top marker transcripts are in orange (e.g., *Hnf4a, Erbb3, Irf1* and *Ceacam1*) (image2) (Additional Files 2: Table S20). c, Heatmap for top 30 apical and basal transcripts in different zones of lIT with log_2_ fold change (Additional Files 2: Table S21). d, Comparison between sIT and lIT by the top 10 apical and basal heatmap of log_2_ fold change (Additional Files 2: Table S18, 21). The genes were selected and aligned based on sIT’s expression profile. Colored dots indicate GO term categories. Blue dot: nutrient sensing and digesting, green dot: apical membrane receptor and receptor binding, purple dot: basal membrane receptor and receptor binding, yellow dot: pathogen defense, red dot: junctional and cytoskeletal category and gray dot: non-categorized. e, UpSet plot to compare between different zones in sIT and lIT and apical to basal, respectively.

Additional files 2: Supplementary Tables 1-27. Table S1. Differential expression analysis results between DPP4 and GFP tethered APEX sequencing. Table S2. Categorized GO terms and Counts for the each categorized GO term. Table S3. GSEA of the APEX-seq data from DE results of DPP4. Table S4. Normalized gene counts of Mitochondria tagged samples. Table S5. Differential gene expression analysis results between ACTB and GFP and GO analysis results for ACTB enriched genes compared to GFP in APEX2 seq data. Table S6. Comparison between Apex2-seq and LCM data from [2]. Table S7. Z-score values of the selected transcripts from APEX sequencing. Table S8. smFISH quantification data using spot detection analysis. Table S9. smFISH quantification data of each ROI from the gradient spot detection analysis. Table S10. Quantification of diameter of RNA granules. Table S11. RBP-MS data comparison between Lct-3'UTR and Fluc. Table S12. RBP-MS data comparison between Net1-3'UTR and Fluc. Table S13. MERFISH 500 gene panel with barcode IDs. Table S14. Volume normalized MERFISH dot count differences between apical and basal sides of sIOs sections. Table S15. Shared gene list from UpSet plot on APEX-seq and sIOs-MERFISH data. Table S16. Wilcoxon test on sIOs-MERFISH data between apical and basal regions per gene. Table S17. Volume normalized MERFISH dot count differences between apical and basal sides of sIT sections. Table S18. Volume normalized MERFISH dot count for whole cell mass for sIT. Table S19. Venn diagram of sIOs and sIT (apical, basal and intersections). Table S20. Volume normalized MERFISH dot count differences between apical and basal sides of lIT sections. Table S21. Volume normalized MERFISH dot count for whole cell mass for lIT. Table S22. The scaled RNA-Protein plot with the GO categorization for villus Top and villus Bottom. Table S23. Plasmid IDs and sequences for APEX-seq and 3'UTR work. Table S24. qPCR results and primer information for mito APEX-seq. Table S25. Primer sequences for in vitro transcription. Table S26. Plasmid IDs and sequences for shRNA work. Table S27. qPCR results and primer information for shRNA work.

## Data Availability

All data presented are available in the main text and supplementary materials. Raw sequencing data, mass spectrometry data, and imaging data can be found: Andreas E. Moor, Minkyoung Lee, Ilhan E. Acar, Davide Eletto, Srivathsan Adivarahan, Farah Mhamedi, Kristina Handler, Jihyun Lee, Elena Guido Vinzoni, Gustavo Aguilar, Systematic discovery of subcellular RNA patterns in the gut epithelium. GEO. https://www.ncbi.nlm.nih.gov/geo/query/acc.cgi with the accession number: GSE267163 (2024). Andreas E. Moor, Minkyoung Lee, Ilhan E. Acar, Davide Eletto, Srivathsan Adivarahan, Farah Mhamedi, Kristina Handler, Jihyun Lee, Elena Guido Vinzoni, Gustavo Aguilar, Systematic discovery of subcellular RNA patterns in the gut epithelium. Figshare. 10.6084/m9.figshare.30257704 (2025).
