# Supplementary figures and images for: Systematic discovery of subcellular RNA patterns in the gut epithelium
Source: Genome Biol. 2025 Oct 29;26:374. doi: 10.1186/s13059-025-03786-1 (PMC12570857; doi:10.1186/s13059-025-03786-1)

## Supplementary Figure 1

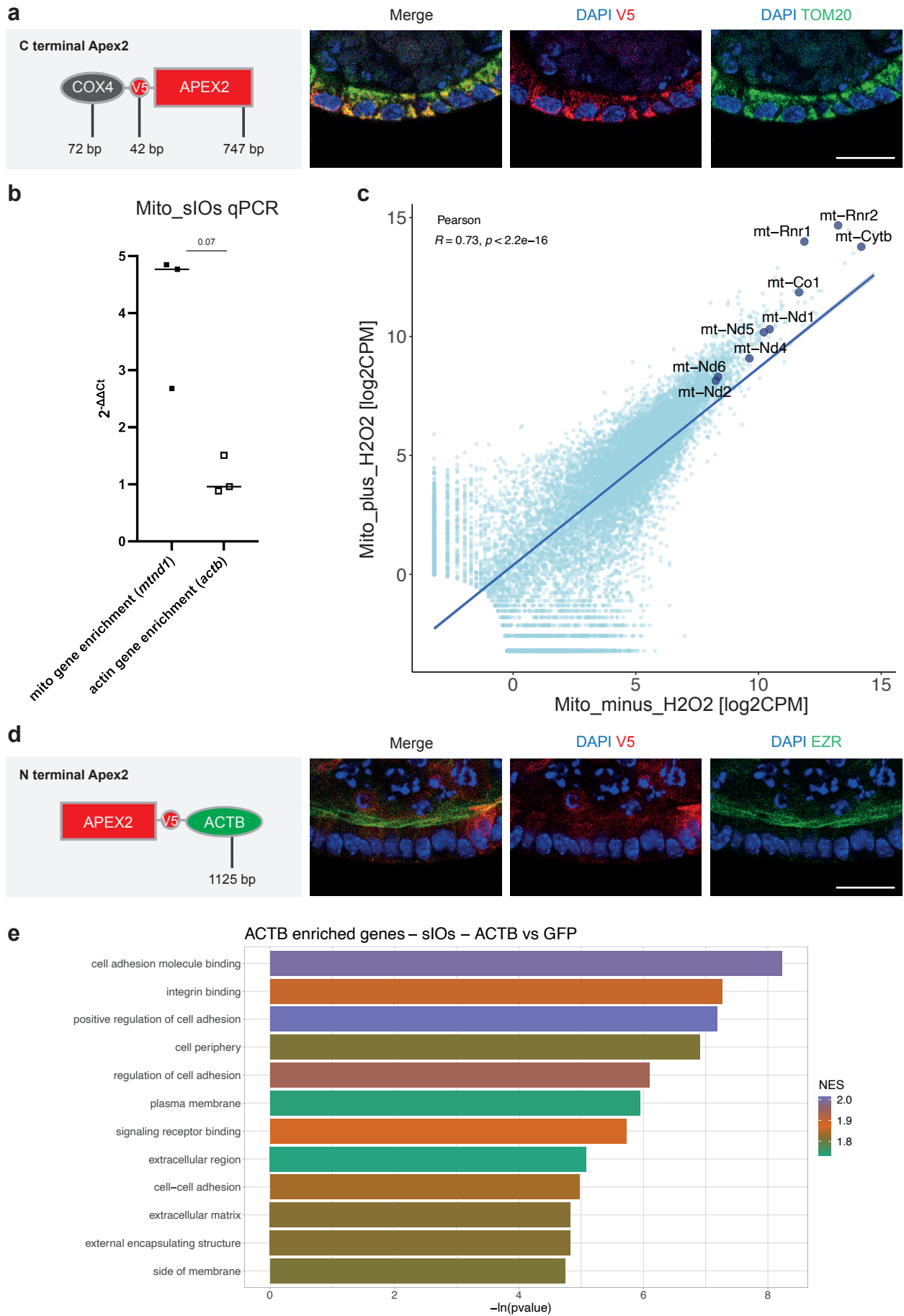

Supplement: Supplementary file 1 — Additional files 1: Supplementary Figs. 1–9. Fig. S1: Proof of concept of APEX-seq in sIOs. a, Left: Scheme of the COX4 construct (also known as MITO-V5-APEX2) used as a bait to capture RNAs localizing inside of mitochondria. Right: Representative immunofluorescence images of COX4-V5-APEX2 expressing organoids. Scale bar 20 µm. b, qPCR results for the mitochondrial RNA, Mt-nd1. Results of ratio paired t test are indicated. Each dot represents one sample. Median indicated as the black bar. c, Correlation plot between Mitochondria samples with and without H2O2. A regression line is fit to log2 transformed counts per million (CPM) normalized counts to show the expression trend of the samples. Highly expressed mitochondrial genes are highlighted with dark blue color and labeled with their names (Additional Files 2: Table S4). d, Left: Scheme of the ACTB construct used as bait to capture RNAs localizing to the cytoplasm. Right: Representative immunofluorescence images of ACTB-V5-APEX2 expressing organoids. Scale bar 20 µm. e, GSEA results of ACTB enriched transcriptome from differential expression analysis using GO terms and normalized enrichment score (NES) (Additional Files 2: Table S5,6). Fig. S2: Quality control of APEX sequencing. a, Schematic representation of the constructs of DPP4 (apical bait) and GFP (cytoplasmic bait) attached to APEX2 machinery. b, Representative fluorescence of sIOs 2 days after lentiviral transduction. Scale bar 200 µm, upper panel and 20 µm, lower panel. c, Representative immunofluorescence images of sIOs stained with Streptavidin-A647 indicating biotinylated molecules in gray. Location of insets indicated with white Dashed boxes. DAPI in blue. Scale bar 20 µm and 5 µm for inset. d, The RNA integrity number (RIN) is shown for different samples. High RNA quality is insured both plus and minus H2O2 application. e, Dot blot with Streptavidin-A680. The biotinylated RNA is detected via staining with streptavidin. f, Representative correlation p [file 13059_2025_3786_MOESM1_ESM.zip › supfig1.pdf]

Supplementary Figure 2

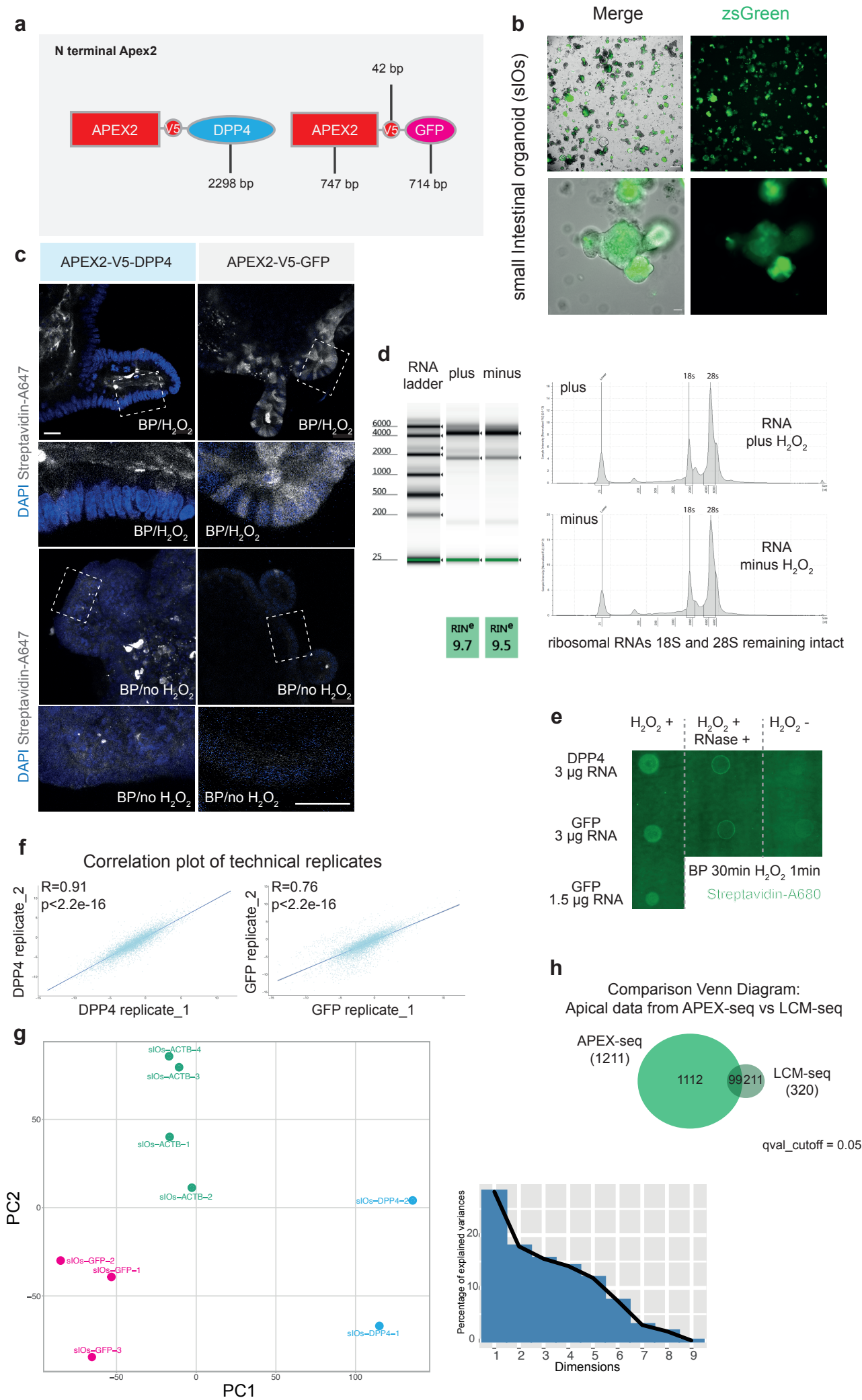

Supplement: Supplementary file 1 — Additional files 1: Supplementary Figs. 1–9. Fig. S1: Proof of concept of APEX-seq in sIOs. a, Left: Scheme of the COX4 construct (also known as MITO-V5-APEX2) used as a bait to capture RNAs localizing inside of mitochondria. Right: Representative immunofluorescence images of COX4-V5-APEX2 expressing organoids. Scale bar 20 µm. b, qPCR results for the mitochondrial RNA, Mt-nd1. Results of ratio paired t test are indicated. Each dot represents one sample. Median indicated as the black bar. c, Correlation plot between Mitochondria samples with and without H2O2. A regression line is fit to log2 transformed counts per million (CPM) normalized counts to show the expression trend of the samples. Highly expressed mitochondrial genes are highlighted with dark blue color and labeled with their names (Additional Files 2: Table S4). d, Left: Scheme of the ACTB construct used as bait to capture RNAs localizing to the cytoplasm. Right: Representative immunofluorescence images of ACTB-V5-APEX2 expressing organoids. Scale bar 20 µm. e, GSEA results of ACTB enriched transcriptome from differential expression analysis using GO terms and normalized enrichment score (NES) (Additional Files 2: Table S5,6). Fig. S2: Quality control of APEX sequencing. a, Schematic representation of the constructs of DPP4 (apical bait) and GFP (cytoplasmic bait) attached to APEX2 machinery. b, Representative fluorescence of sIOs 2 days after lentiviral transduction. Scale bar 200 µm, upper panel and 20 µm, lower panel. c, Representative immunofluorescence images of sIOs stained with Streptavidin-A647 indicating biotinylated molecules in gray. Location of insets indicated with white Dashed boxes. DAPI in blue. Scale bar 20 µm and 5 µm for inset. d, The RNA integrity number (RIN) is shown for different samples. High RNA quality is insured both plus and minus H2O2 application. e, Dot blot with Streptavidin-A680. The biotinylated RNA is detected via staining with streptavidin. f, Representative correlation p [file 13059_2025_3786_MOESM1_ESM.zip › supfig2.pdf]

Supplementary Figure 3

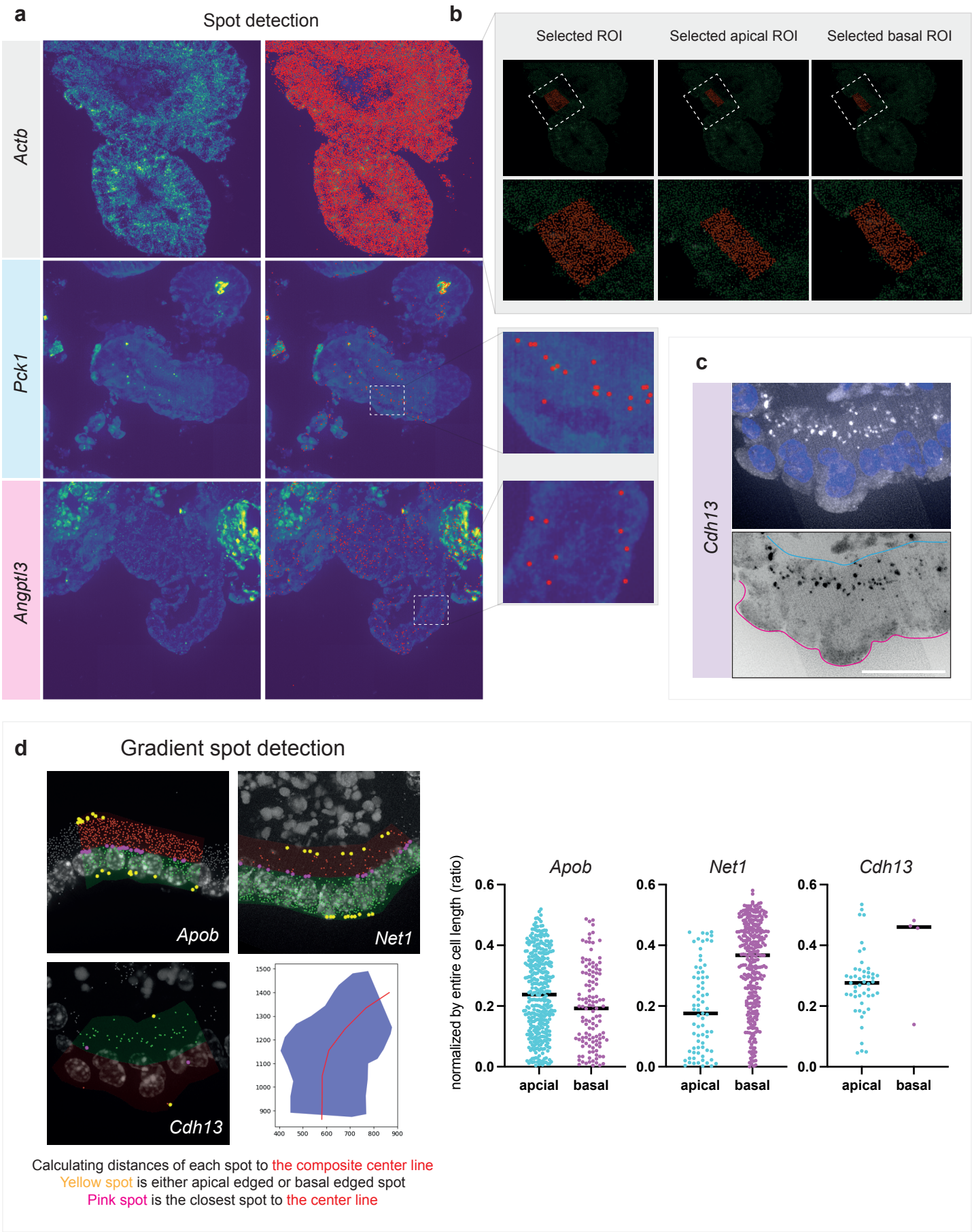

Supplement: Supplementary file 1 — Additional files 1: Supplementary Figs. 1–9. Fig. S1: Proof of concept of APEX-seq in sIOs. a, Left: Scheme of the COX4 construct (also known as MITO-V5-APEX2) used as a bait to capture RNAs localizing inside of mitochondria. Right: Representative immunofluorescence images of COX4-V5-APEX2 expressing organoids. Scale bar 20 µm. b, qPCR results for the mitochondrial RNA, Mt-nd1. Results of ratio paired t test are indicated. Each dot represents one sample. Median indicated as the black bar. c, Correlation plot between Mitochondria samples with and without H2O2. A regression line is fit to log2 transformed counts per million (CPM) normalized counts to show the expression trend of the samples. Highly expressed mitochondrial genes are highlighted with dark blue color and labeled with their names (Additional Files 2: Table S4). d, Left: Scheme of the ACTB construct used as bait to capture RNAs localizing to the cytoplasm. Right: Representative immunofluorescence images of ACTB-V5-APEX2 expressing organoids. Scale bar 20 µm. e, GSEA results of ACTB enriched transcriptome from differential expression analysis using GO terms and normalized enrichment score (NES) (Additional Files 2: Table S5,6). Fig. S2: Quality control of APEX sequencing. a, Schematic representation of the constructs of DPP4 (apical bait) and GFP (cytoplasmic bait) attached to APEX2 machinery. b, Representative fluorescence of sIOs 2 days after lentiviral transduction. Scale bar 200 µm, upper panel and 20 µm, lower panel. c, Representative immunofluorescence images of sIOs stained with Streptavidin-A647 indicating biotinylated molecules in gray. Location of insets indicated with white Dashed boxes. DAPI in blue. Scale bar 20 µm and 5 µm for inset. d, The RNA integrity number (RIN) is shown for different samples. High RNA quality is insured both plus and minus H2O2 application. e, Dot blot with Streptavidin-A680. The biotinylated RNA is detected via staining with streptavidin. f, Representative correlation p [file 13059_2025_3786_MOESM1_ESM.zip › supfig3.pdf]

Supplementary Figure 4

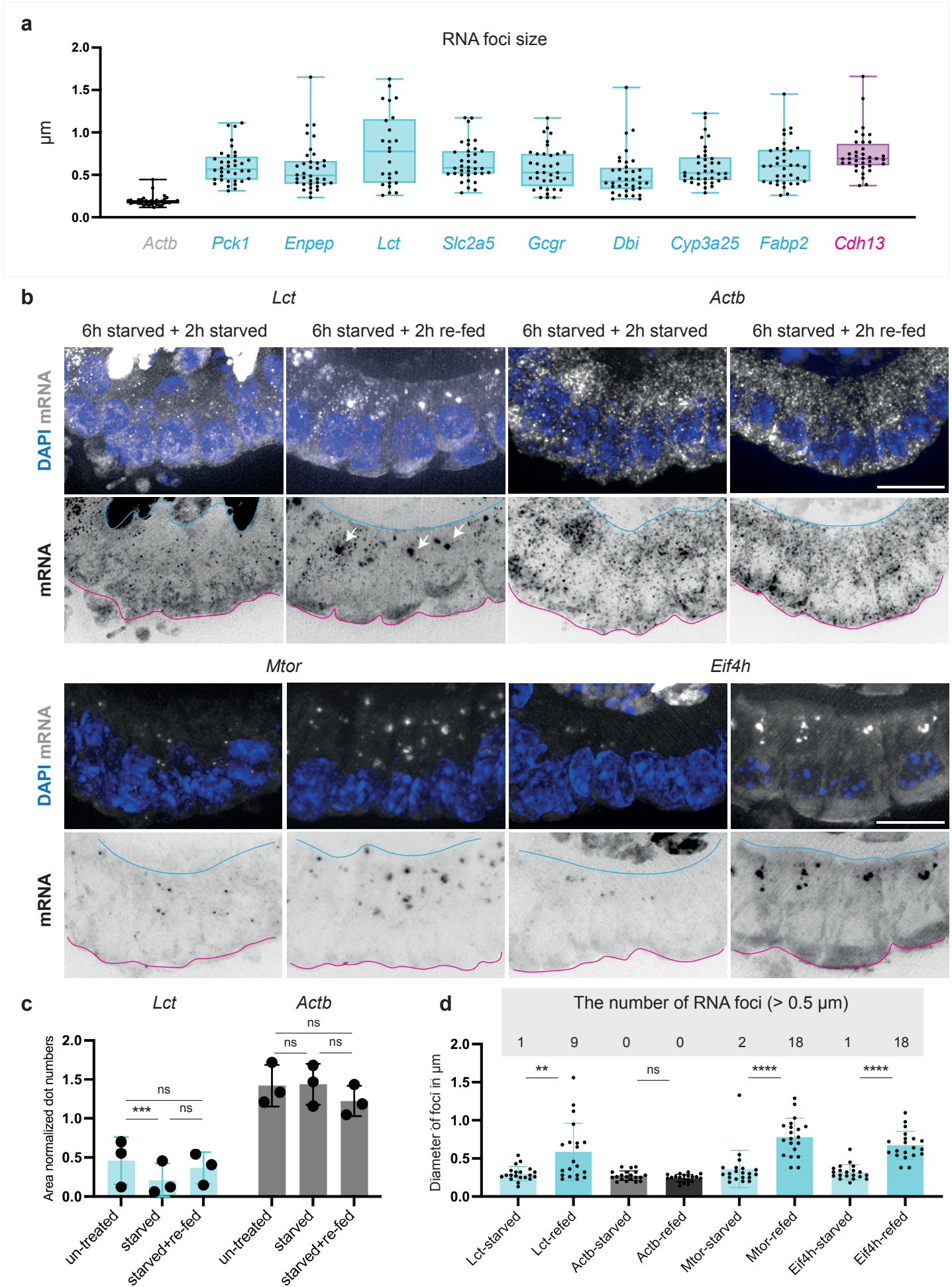

Supplement: Supplementary file 1 — Additional files 1: Supplementary Figs. 1–9. Fig. S1: Proof of concept of APEX-seq in sIOs. a, Left: Scheme of the COX4 construct (also known as MITO-V5-APEX2) used as a bait to capture RNAs localizing inside of mitochondria. Right: Representative immunofluorescence images of COX4-V5-APEX2 expressing organoids. Scale bar 20 µm. b, qPCR results for the mitochondrial RNA, Mt-nd1. Results of ratio paired t test are indicated. Each dot represents one sample. Median indicated as the black bar. c, Correlation plot between Mitochondria samples with and without H2O2. A regression line is fit to log2 transformed counts per million (CPM) normalized counts to show the expression trend of the samples. Highly expressed mitochondrial genes are highlighted with dark blue color and labeled with their names (Additional Files 2: Table S4). d, Left: Scheme of the ACTB construct used as bait to capture RNAs localizing to the cytoplasm. Right: Representative immunofluorescence images of ACTB-V5-APEX2 expressing organoids. Scale bar 20 µm. e, GSEA results of ACTB enriched transcriptome from differential expression analysis using GO terms and normalized enrichment score (NES) (Additional Files 2: Table S5,6). Fig. S2: Quality control of APEX sequencing. a, Schematic representation of the constructs of DPP4 (apical bait) and GFP (cytoplasmic bait) attached to APEX2 machinery. b, Representative fluorescence of sIOs 2 days after lentiviral transduction. Scale bar 200 µm, upper panel and 20 µm, lower panel. c, Representative immunofluorescence images of sIOs stained with Streptavidin-A647 indicating biotinylated molecules in gray. Location of insets indicated with white Dashed boxes. DAPI in blue. Scale bar 20 µm and 5 µm for inset. d, The RNA integrity number (RIN) is shown for different samples. High RNA quality is insured both plus and minus H2O2 application. e, Dot blot with Streptavidin-A680. The biotinylated RNA is detected via staining with streptavidin. f, Representative correlation p [file 13059_2025_3786_MOESM1_ESM.zip › supfig4.pdf]

Supplementary Figure 5

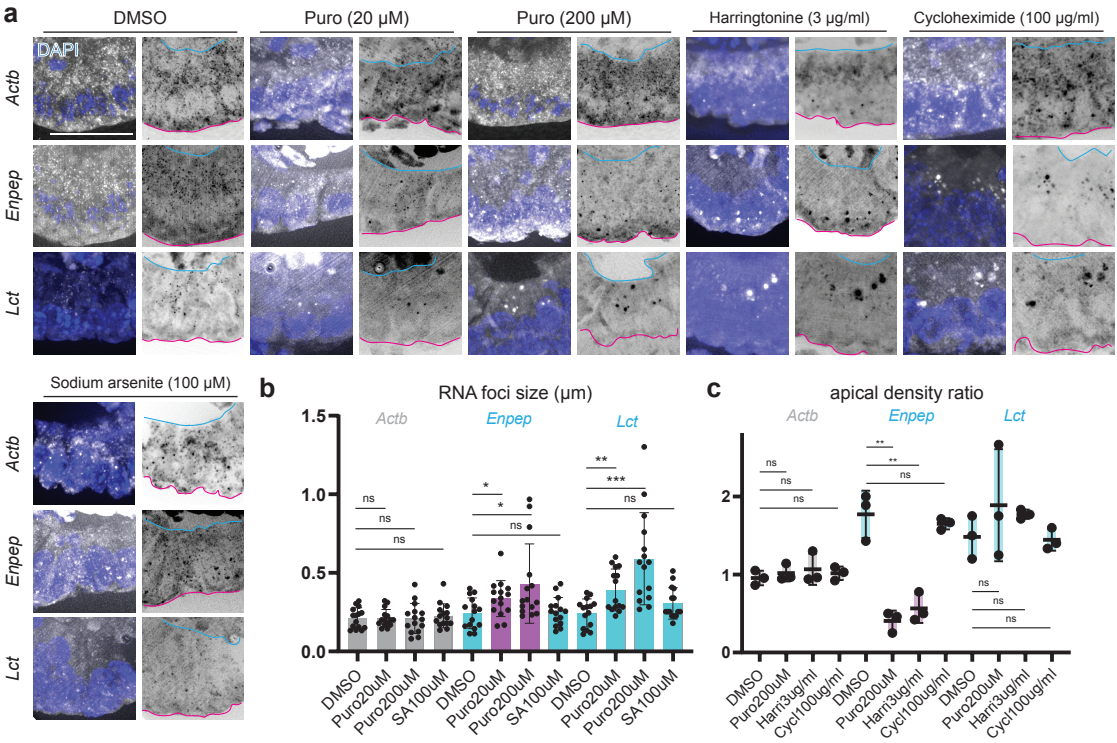

Supplement: Supplementary file 1 — Additional files 1: Supplementary Figs. 1–9. Fig. S1: Proof of concept of APEX-seq in sIOs. a, Left: Scheme of the COX4 construct (also known as MITO-V5-APEX2) used as a bait to capture RNAs localizing inside of mitochondria. Right: Representative immunofluorescence images of COX4-V5-APEX2 expressing organoids. Scale bar 20 µm. b, qPCR results for the mitochondrial RNA, Mt-nd1. Results of ratio paired t test are indicated. Each dot represents one sample. Median indicated as the black bar. c, Correlation plot between Mitochondria samples with and without H2O2. A regression line is fit to log2 transformed counts per million (CPM) normalized counts to show the expression trend of the samples. Highly expressed mitochondrial genes are highlighted with dark blue color and labeled with their names (Additional Files 2: Table S4). d, Left: Scheme of the ACTB construct used as bait to capture RNAs localizing to the cytoplasm. Right: Representative immunofluorescence images of ACTB-V5-APEX2 expressing organoids. Scale bar 20 µm. e, GSEA results of ACTB enriched transcriptome from differential expression analysis using GO terms and normalized enrichment score (NES) (Additional Files 2: Table S5,6). Fig. S2: Quality control of APEX sequencing. a, Schematic representation of the constructs of DPP4 (apical bait) and GFP (cytoplasmic bait) attached to APEX2 machinery. b, Representative fluorescence of sIOs 2 days after lentiviral transduction. Scale bar 200 µm, upper panel and 20 µm, lower panel. c, Representative immunofluorescence images of sIOs stained with Streptavidin-A647 indicating biotinylated molecules in gray. Location of insets indicated with white Dashed boxes. DAPI in blue. Scale bar 20 µm and 5 µm for inset. d, The RNA integrity number (RIN) is shown for different samples. High RNA quality is insured both plus and minus H2O2 application. e, Dot blot with Streptavidin-A680. The biotinylated RNA is detected via staining with streptavidin. f, Representative correlation p [file 13059_2025_3786_MOESM1_ESM.zip › supfig5.pdf]

Supplementary Figure 6

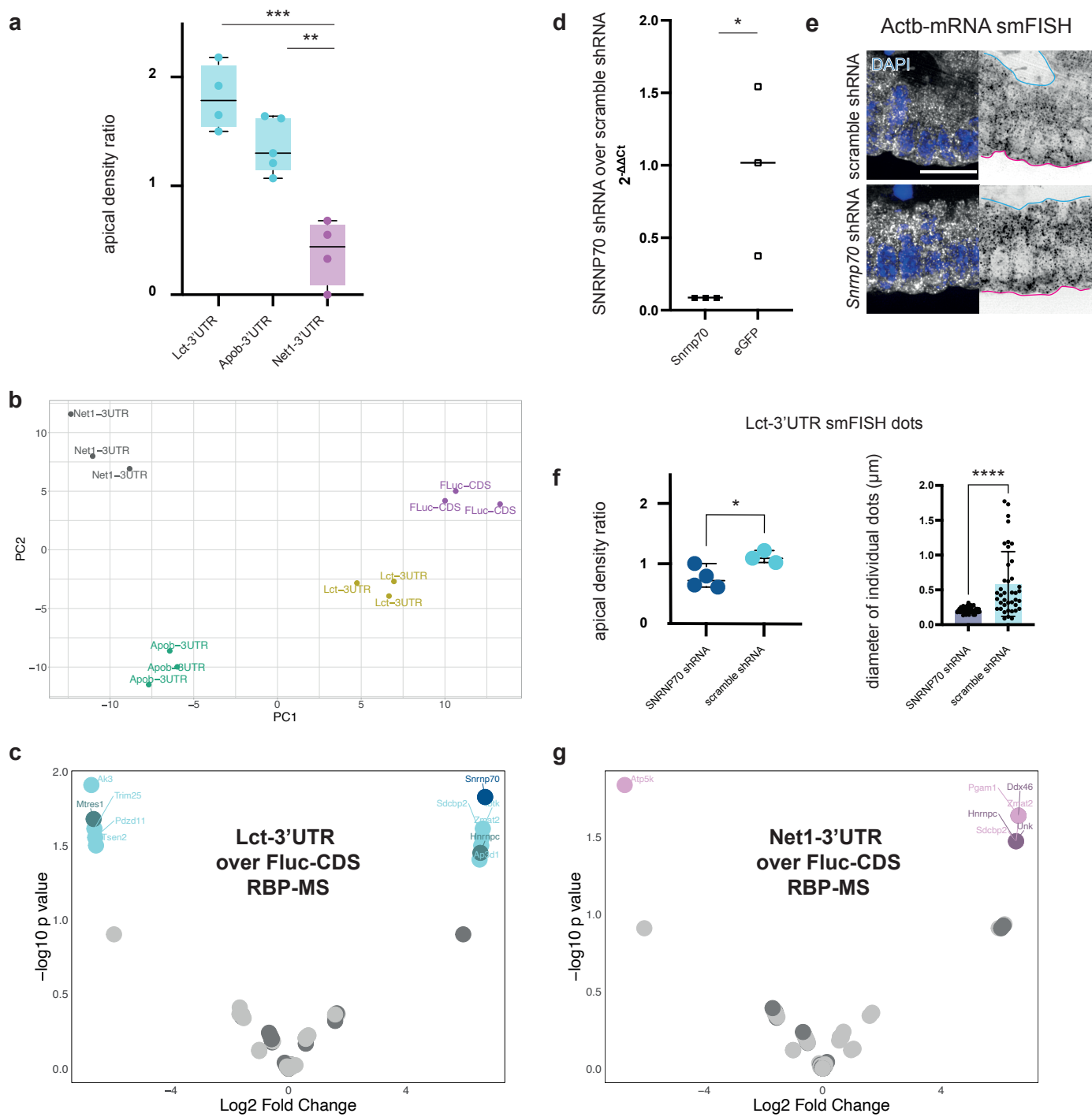

Supplement: Supplementary file 1 — Additional files 1: Supplementary Figs. 1–9. Fig. S1: Proof of concept of APEX-seq in sIOs. a, Left: Scheme of the COX4 construct (also known as MITO-V5-APEX2) used as a bait to capture RNAs localizing inside of mitochondria. Right: Representative immunofluorescence images of COX4-V5-APEX2 expressing organoids. Scale bar 20 µm. b, qPCR results for the mitochondrial RNA, Mt-nd1. Results of ratio paired t test are indicated. Each dot represents one sample. Median indicated as the black bar. c, Correlation plot between Mitochondria samples with and without H2O2. A regression line is fit to log2 transformed counts per million (CPM) normalized counts to show the expression trend of the samples. Highly expressed mitochondrial genes are highlighted with dark blue color and labeled with their names (Additional Files 2: Table S4). d, Left: Scheme of the ACTB construct used as bait to capture RNAs localizing to the cytoplasm. Right: Representative immunofluorescence images of ACTB-V5-APEX2 expressing organoids. Scale bar 20 µm. e, GSEA results of ACTB enriched transcriptome from differential expression analysis using GO terms and normalized enrichment score (NES) (Additional Files 2: Table S5,6). Fig. S2: Quality control of APEX sequencing. a, Schematic representation of the constructs of DPP4 (apical bait) and GFP (cytoplasmic bait) attached to APEX2 machinery. b, Representative fluorescence of sIOs 2 days after lentiviral transduction. Scale bar 200 µm, upper panel and 20 µm, lower panel. c, Representative immunofluorescence images of sIOs stained with Streptavidin-A647 indicating biotinylated molecules in gray. Location of insets indicated with white Dashed boxes. DAPI in blue. Scale bar 20 µm and 5 µm for inset. d, The RNA integrity number (RIN) is shown for different samples. High RNA quality is insured both plus and minus H2O2 application. e, Dot blot with Streptavidin-A680. The biotinylated RNA is detected via staining with streptavidin. f, Representative correlation p [file 13059_2025_3786_MOESM1_ESM.zip › supfig6.pdf]

Supplementary Figure 7

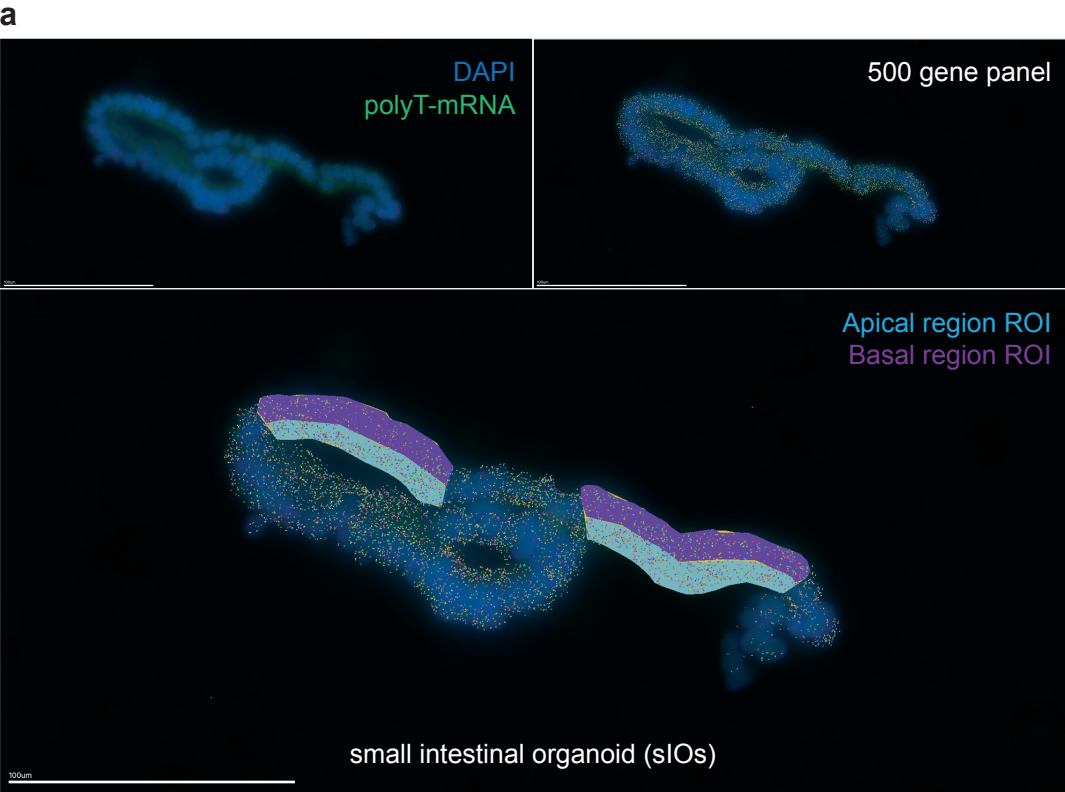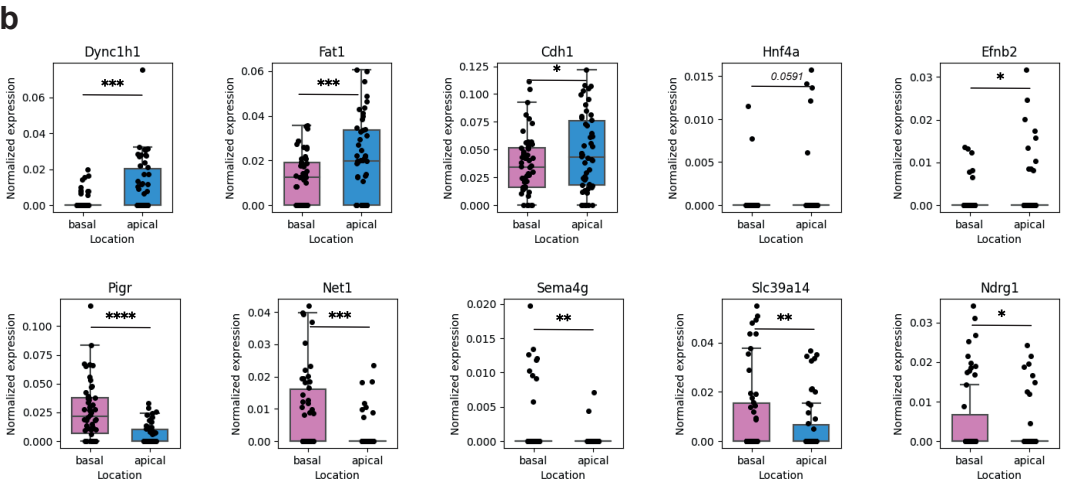

Supplement: Supplementary file 1 — Additional files 1: Supplementary Figs. 1–9. Fig. S1: Proof of concept of APEX-seq in sIOs. a, Left: Scheme of the COX4 construct (also known as MITO-V5-APEX2) used as a bait to capture RNAs localizing inside of mitochondria. Right: Representative immunofluorescence images of COX4-V5-APEX2 expressing organoids. Scale bar 20 µm. b, qPCR results for the mitochondrial RNA, Mt-nd1. Results of ratio paired t test are indicated. Each dot represents one sample. Median indicated as the black bar. c, Correlation plot between Mitochondria samples with and without H2O2. A regression line is fit to log2 transformed counts per million (CPM) normalized counts to show the expression trend of the samples. Highly expressed mitochondrial genes are highlighted with dark blue color and labeled with their names (Additional Files 2: Table S4). d, Left: Scheme of the ACTB construct used as bait to capture RNAs localizing to the cytoplasm. Right: Representative immunofluorescence images of ACTB-V5-APEX2 expressing organoids. Scale bar 20 µm. e, GSEA results of ACTB enriched transcriptome from differential expression analysis using GO terms and normalized enrichment score (NES) (Additional Files 2: Table S5,6). Fig. S2: Quality control of APEX sequencing. a, Schematic representation of the constructs of DPP4 (apical bait) and GFP (cytoplasmic bait) attached to APEX2 machinery. b, Representative fluorescence of sIOs 2 days after lentiviral transduction. Scale bar 200 µm, upper panel and 20 µm, lower panel. c, Representative immunofluorescence images of sIOs stained with Streptavidin-A647 indicating biotinylated molecules in gray. Location of insets indicated with white Dashed boxes. DAPI in blue. Scale bar 20 µm and 5 µm for inset. d, The RNA integrity number (RIN) is shown for different samples. High RNA quality is insured both plus and minus H2O2 application. e, Dot blot with Streptavidin-A680. The biotinylated RNA is detected via staining with streptavidin. f, Representative correlation p [file 13059_2025_3786_MOESM1_ESM.zip › supfig7.pdf]

Supplementary Figure 8

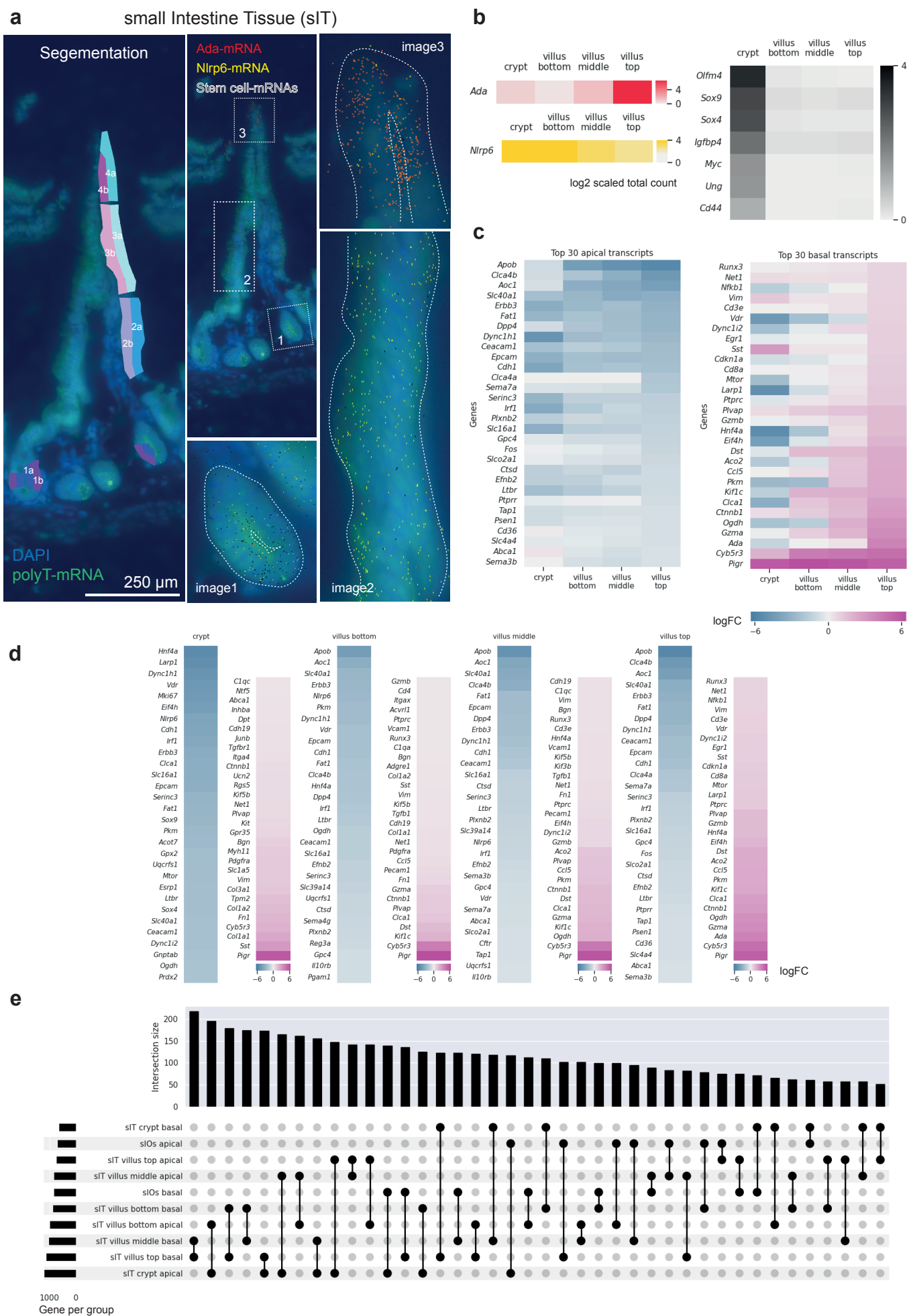

Supplement: Supplementary file 1 — Additional files 1: Supplementary Figs. 1–9. Fig. S1: Proof of concept of APEX-seq in sIOs. a, Left: Scheme of the COX4 construct (also known as MITO-V5-APEX2) used as a bait to capture RNAs localizing inside of mitochondria. Right: Representative immunofluorescence images of COX4-V5-APEX2 expressing organoids. Scale bar 20 µm. b, qPCR results for the mitochondrial RNA, Mt-nd1. Results of ratio paired t test are indicated. Each dot represents one sample. Median indicated as the black bar. c, Correlation plot between Mitochondria samples with and without H2O2. A regression line is fit to log2 transformed counts per million (CPM) normalized counts to show the expression trend of the samples. Highly expressed mitochondrial genes are highlighted with dark blue color and labeled with their names (Additional Files 2: Table S4). d, Left: Scheme of the ACTB construct used as bait to capture RNAs localizing to the cytoplasm. Right: Representative immunofluorescence images of ACTB-V5-APEX2 expressing organoids. Scale bar 20 µm. e, GSEA results of ACTB enriched transcriptome from differential expression analysis using GO terms and normalized enrichment score (NES) (Additional Files 2: Table S5,6). Fig. S2: Quality control of APEX sequencing. a, Schematic representation of the constructs of DPP4 (apical bait) and GFP (cytoplasmic bait) attached to APEX2 machinery. b, Representative fluorescence of sIOs 2 days after lentiviral transduction. Scale bar 200 µm, upper panel and 20 µm, lower panel. c, Representative immunofluorescence images of sIOs stained with Streptavidin-A647 indicating biotinylated molecules in gray. Location of insets indicated with white Dashed boxes. DAPI in blue. Scale bar 20 µm and 5 µm for inset. d, The RNA integrity number (RIN) is shown for different samples. High RNA quality is insured both plus and minus H2O2 application. e, Dot blot with Streptavidin-A680. The biotinylated RNA is detected via staining with streptavidin. f, Representative correlation p [file 13059_2025_3786_MOESM1_ESM.zip › supfig8.pdf]

Supplementary Figure 9

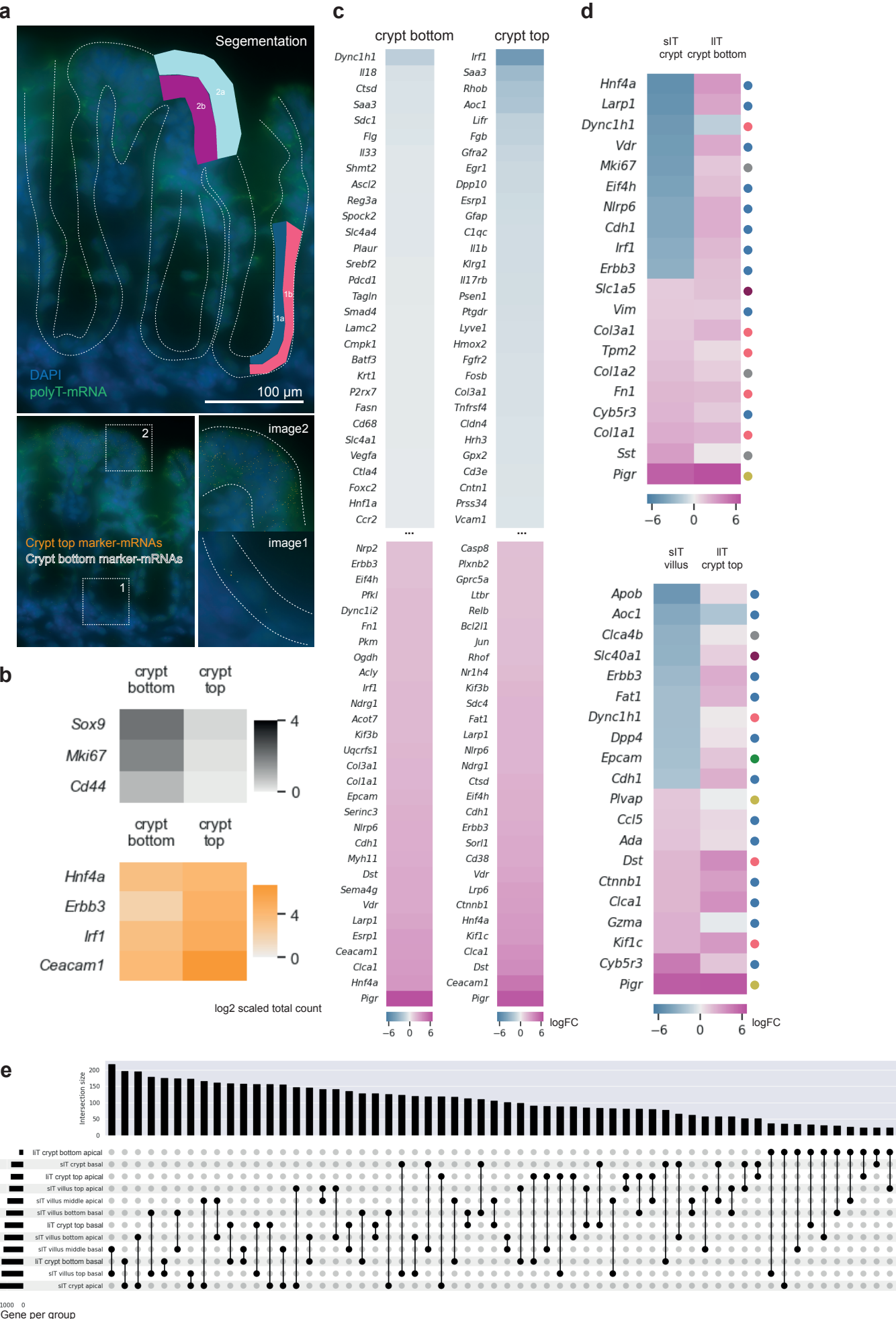

Supplement: Supplementary file 1 — Additional files 1: Supplementary Figs. 1–9. Fig. S1: Proof of concept of APEX-seq in sIOs. a, Left: Scheme of the COX4 construct (also known as MITO-V5-APEX2) used as a bait to capture RNAs localizing inside of mitochondria. Right: Representative immunofluorescence images of COX4-V5-APEX2 expressing organoids. Scale bar 20 µm. b, qPCR results for the mitochondrial RNA, Mt-nd1. Results of ratio paired t test are indicated. Each dot represents one sample. Median indicated as the black bar. c, Correlation plot between Mitochondria samples with and without H2O2. A regression line is fit to log2 transformed counts per million (CPM) normalized counts to show the expression trend of the samples. Highly expressed mitochondrial genes are highlighted with dark blue color and labeled with their names (Additional Files 2: Table S4). d, Left: Scheme of the ACTB construct used as bait to capture RNAs localizing to the cytoplasm. Right: Representative immunofluorescence images of ACTB-V5-APEX2 expressing organoids. Scale bar 20 µm. e, GSEA results of ACTB enriched transcriptome from differential expression analysis using GO terms and normalized enrichment score (NES) (Additional Files 2: Table S5,6). Fig. S2: Quality control of APEX sequencing. a, Schematic representation of the constructs of DPP4 (apical bait) and GFP (cytoplasmic bait) attached to APEX2 machinery. b, Representative fluorescence of sIOs 2 days after lentiviral transduction. Scale bar 200 µm, upper panel and 20 µm, lower panel. c, Representative immunofluorescence images of sIOs stained with Streptavidin-A647 indicating biotinylated molecules in gray. Location of insets indicated with white Dashed boxes. DAPI in blue. Scale bar 20 µm and 5 µm for inset. d, The RNA integrity number (RIN) is shown for different samples. High RNA quality is insured both plus and minus H2O2 application. e, Dot blot with Streptavidin-A680. The biotinylated RNA is detected via staining with streptavidin. f, Representative correlation p [file 13059_2025_3786_MOESM1_ESM.zip › supfig9.pdf]
